# Single-cell microfluidics facilitates the rapid quantification of antibiotic accumulation in Gram-negative bacteria†
†Electronic supplementary information (ESI) available: All the data is available in the main text or in the ESI. See DOI: 10.1039/d0lc00242a


**DOI:** 10.1039/d0lc00242a

**Published:** 2020-06-16

**Authors:** Jehangir Cama, Margaritis Voliotis, Jeremy Metz, Ashley Smith, Jari Iannucci, Ulrich F. Keyser, Krasimira Tsaneva-Atanasova, Stefano Pagliara

**Affiliations:** a Living Systems Institute , University of Exeter , Exeter EX4 4QD , UK . Email: j.cama@exeter.ac.uk; b College of Engineering , Mathematics and Physical Sciences , University of Exeter , Exeter EX4 4QF , UK; c Cavendish Laboratory , Department of Physics , University of Cambridge , JJ Thomson Avenue , Cambridge CB3 0HE , UK; d School of Biosciences , College of Life and Environmental Sciences , University of Exeter , Exeter EX4 4QD , UK . Email: s.pagliara@exeter.ac.uk

## Abstract

The double-membrane cell envelope of Gram-negative bacteria is a formidable barrier to intracellular antibiotic accumulation. A quantitative understanding of antibiotic transport in these cells is crucial for drug development, but this has proved elusive due to a dearth of suitable investigative techniques. Here we combine microfluidics and time-lapse auto-fluorescence microscopy to rapidly quantify antibiotic accumulation in hundreds of individual *Escherichia coli* cells. By serially manipulating the microfluidic environment, we demonstrated that stationary phase *Escherichia coli*, traditionally more refractory to antibiotics than growing cells, display reduced accumulation of the antibiotic ofloxacin compared to actively growing cells. Our novel microfluidic method facilitates the quantitative comparison of the role of the microenvironment *versus* that of the absence of key membrane transport pathways in cellular drug accumulation. Unlike traditional techniques, our assay is rapid, studying accumulation as the cells are dosed with the drug. This platform provides a powerful new tool for studying antibiotic accumulation in bacteria, which will be critical for the rational development of the next generation of antibiotics.

## Abstract 2

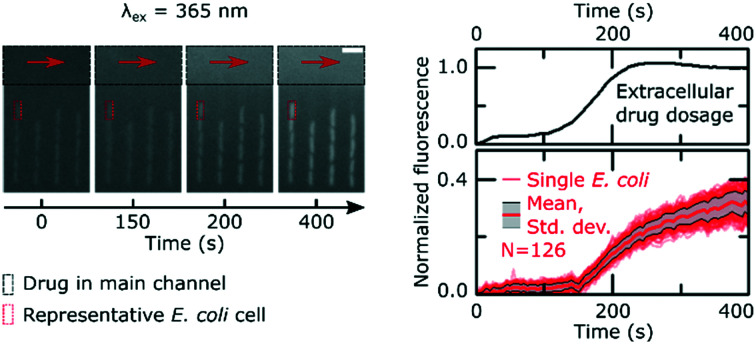
A novel, rapid single-cell assay for quantifying antibiotic accumulation in Gram-negative bacteria reveals important insights about bacterial drug accumulation.

## Introduction

Antimicrobial resistance is recognized as a major global health threat, and treatment failures in patients with microbial infections are predicted to cause 10 million deaths annually by 2050.^1^ There is thus a desperate need to refresh the antibiotic development pipeline and develop new technologies to optimize antibiotic treatment. Infections caused by Gram-negative bacteria are of particular concern, due to the protection against antibiotics provided by the complex double-membrane cell envelopes of these organisms. These bacteria display an asymmetric outer membrane that contains lipopolysaccharide (LPS) molecules, which create a formidable permeability barrier to the cellular entry of both hydrophilic and hydrophobic molecules.^2^,^3^ Antibiotic permeation across the outer membrane is therefore dependent on the drug's ability to utilize protein pores (or *porins*),^4^–^6^ typically used for nutrient uptake, to circumvent this barrier. These porins show a preference for hydrophilic, charged compounds; however, antibiotics that are active against targets located in the cytoplasm have to also cross the inner membrane phospholipid bilayer, which acts as a selectivity barrier *against* polar, charged molecules.^2^,^3^ Additionally, Gram-negative bacteria harbor active efflux mechanisms, which pump toxic compounds out of the cell.^7^ Successful drugs must minimize their propensity for recognition and removal by these efflux pumps, in addition to displaying specific physicochemical properties to permeate both through the outer membrane porins and inner membrane phospholipids.^2^ Crucially, the expression and activity of porins and efflux pumps vary i) with microenvironment conditions^8^ and ii) within bacteria with the same genetic make-up that are exposed to similar environments.^9^ Quantitative methods for studying drug accumulation in individual bacteria are therefore crucial for understanding this molecular transport landscape, to drive the rational development of the next generation of antibiotics.^2^,^3^

However, the most commonly used techniques to study antibiotic accumulation in bacteria are population level assays which cannot investigate accumulation at the single-cell level; thus heterogeneity in transport pathways between individual cells, and any downstream effects on antibiotic survival are not resolvable with the majority of current techniques. Perhaps even more importantly, most existing experimental techniques suffer from the requirement of complex washing steps,^3^,^10^ with cells only studied after resuspension in contrived nutrient environments^11^,^12^; the washes increase the chance of cell lysis and efflux or diffusion of the analyte from the cells,^3^ besides affecting cellular physiology. Most available techniques further only provide a static picture rather than the dynamics of drug accumulation.

There is therefore a need to fundamentally change the experimental approach for quantifying antibiotic accumulation in bacteria, to incorporate single-cell level methodologies and the ability to study drug accumulation after exposure to different nutrient conditions or in different metabolic states. Ideally, this approach should also be simple to implement to ensure its uptake in pharmaceutical companies and in clinical settings.

Here, we address these myriad challenges by harnessing the power of microfluidics and auto-fluorescence microscopy to study the accumulation of the fluoroquinolone antibiotic ofloxacin in up to hundreds of individual bacteria confined in well-defined microenvironments. We require only a small aliquot of bacterial culture to seed a microfluidic “mother-machine” device^13^,^14^ wherein we accurately dose cells either in stationary or in a growing phase with the antibiotic. We simultaneously image ofloxacin accumulation in individual *Escherichia coli* in real-time using the auto-fluorescence of the drug. Since the antibiotic is itself fluorescent, we are able to track both drug dosage (ESI† Note 1) and cellular accumulation in a label-free manner, without influencing the transport properties of the molecule. A number of antibiotics from the fluoroquinolone^15^ and tetracycline^16^,^17^ classes are known to be auto-fluorescent, and recently this property has also been observed in a new class of antibiotics.^18^ Furthermore, in our technique there are no lysis or washing steps post drug delivery, which circumvents the technical challenges associated with the typical post-dosage handling steps described above.

We validated our technique by studying ofloxacin accumulation in three *E. coli* strains from the Keio collection,^19^ encompassing the wild type (WT) BW25113, an OmpF porin knockout (Δ*ompF*) and a TolC efflux protein knockout (Δ*tolC*) strain. It is noteworthy that our methodology enables, for what we believe is the first time, the direct, quantitative comparison of the role of growth phase *versus* the role of the presence of individual transport pathways in drug accumulation. We also complement our experimental single-cell level data with a mathematical model and Bayesian inference to extract details of the kinetics of the transport process in individual cells.

Besides its applications in drug development, our microfluidic platform can complement recently developed lab-on-chip antibiotic susceptibility testing systems to determine the contribution of reduced drug accumulation to bacterial survival in clinical settings.^20^ Furthermore, although this study focuses on Gram-negative bacteria, the experimental and theoretical framework that we employ may be repurposed, with appropriate modifications, for advancing our understanding of molecular transport in a range of fundamental phenomena in both cellular and synthetic systems. This will pave the way for a direct, quantitative evaluation of the role of growth phases, nutrient conditions and transport pathway expression in drug accumulation in a range of cells.

## Experimental

### Chemicals

Chemicals were purchased from Sigma-Aldrich unless otherwise stated. Ofloxacin stock solutions were prepared at a concentration of 10 mg ml^–1^ in 1 M NaOH. For the ofloxacin accumulation experiments, the stock was diluted to a concentration of 12.5 μg ml^–1^ (100 × MIC) in PBS (Phosphate-buffered saline). We chose to always dissolve the ofloxacin in PBS to ensure that the pH conditions remained uniform during drug exposure across all experiments and metabolic conditions; it is well known that pH regulates the charge state of fluoroquinolones, which affects their membrane permeabilities.^15^,^21^ The minimal media used in the experiments was prepared in sterile water and contained 1 × M9 salts, 2 mM MgSO_4_, 0.1 mM CaCl_2_ and 1 mg L^–1^ thiamine hydrochloride. The LB (Lysogeny broth) medium used for cell culture was the Melford high salt version containing 10 g L^–1^ casein digest peptone, 5 g L^–1^ yeast extract and 10 g L^–1^ NaCl; LB Agar plates were prepared with 15 g L^–1^ agar. Glucose stock solutions were prepared at a concentration of 0.5 M in sterile water and diluted to 1 g L^–1^ in minimal media for use in the experiments. Stock solutions of bovine serum albumin (BSA) were prepared at a concentration of 50 mg ml^–1^ in sterile water. A stock solution of propidium iodide (PI) was purchased from Thermo Fisher Scientific, and diluted 1 : 1000 in PBS for use in the experiments.

### Bacterial cell culture

All the *E. coli* strains used were BW25113 strains purchased from the Keio collection. The mutant strains contained kanamycin resistance cassettes in place of the deleted chromosomal gene. The strains were stored at –80 °C in a 1 : 1 ratio of overnight culture and 50% glycerol solution. 200 ml cultures were grown in LB (with 25 μg ml^–1^ kanamycin as necessary) at 37 °C overnight (with shaking at 200 rpm). Streak plates were prepared on LB agar (containing 25 μg ml^–1^ kanamycin as necessary), stored at 4 °C and used for a maximum of one week.

### Microfluidic chip fabrication and cell loading

The complete protocol for the fabrication of the “mother-machine” microfluidic devices was reported previously; these are two-layer microfluidic devices consisting of a “main” seeding channel (height 25 μm and width 100 μm) and thousands of narrower “side-channels” (length 25 μm, height and width 1.4 μm) for confining bacteria.^22^ The volume of the microfluidic channel network (excluding the inlet and outlet columns) in the chip is on the order of 150 nl. The epoxy mold used was constructed from replicas of devices kindly provided by the Jun lab.^14^ The final devices used were created by pouring polydimethylsiloxane (PDMS, Dow Corning, 9 : 1 base: curing agent) on to the epoxy mold; the PDMS was baked at 70 °C for 2 h in an oven. The PDMS chips were cut out and fluidic inlet/outlet columns punched using a 1.5 mm biopsy punch (Miltex); chip heights of around 0.5–1 cm lead to inlet and outlet column volumes of approximately 9–18 μl each. The PDMS chips were bonded to a type 1 coverslip using an air plasma treatment (10 s exposure at 30 W plasma power, Plasma etcher, Diener electronic GmbH, Germany) and left at 70 °C for 5 min to improve the adhesion. The chips were then filled with a 50 mg ml^–1^ solution of bovine serum albumin (BSA, in MilliQ water) and incubated at 37 °C for 1 h. The BSA treatment passivates the internal surfaces of the chip thus preventing cells from adhering to the microchannels during experiments.

An overnight culture of cells (OD_595_ typically between 4.5–5) was resuspended in spent LB and concentrated to an OD of 50 (at 595 nm). The spent LB was prepared by centrifuging the overnight culture (10 min at 3000 g and 20 °C) – the supernatant was filtered twice through a 0.2 μm pore filter (Millipore). An aliquot of this culture solution was injected into the microfluidic device and incubated at 37 °C for 20 min, enabling cells to enter the small side channels of the device. The filled device was then left overnight at room temperature (with the inlet and outlet access ports sealed to prevent any evaporation) before starting experiments.

### Microfluidic pumps

Microfluidic flows were controlled using three parallel neMESYS syringe pumps (Cetoni GmbH, Germany) with glass syringes (ILS, Germany) of volumes 5 ml, 250 μl and 100 μl respectively. The syringes were interfaced with the microfluidic chips using FEP tubing (Upchurch Scientific 1520, inner diameter = 0.03′′ and outer diameter = 0.0625′′). The syringes and the associated tubing were rinsed thoroughly with MilliQ water and the appropriate experimental solutions before beginning the experiments, and with 70% ethanol after completion of the experiments.

### Microscopy setup

All the experiments were performed on an Olympus IX73 epifluorescence microscope with an LED light source (wLS pE300, QImaging) using a 365 nm excitation wavelength LED. The LED was triggered by the camera (Evolve 512 EMCCD, Photometrics) to ensure that the cells were only exposed to the excitation light during image acquisition. The camera was controlled using μManager 1.4.^23^ Fluorescence images were acquired using 10 ms exposure times and an EM gain of 200 (bin 1, clearing mode – pre-exposure). A standard DAPI filter set (Chroma ET series) modified with a ZET 365/20× excitation filter (Chroma) was used to better match the 365 nm excitation wavelength. An Olympus UPLSAPO 60× W (N.A 1.2) objective was used for all the experiments. We used a heating stage (Linkam Scientific THL60-16, UK) to maintain the cells at 37 °C throughout the experiments.

### Drug accumulation assay

For both stationary phase and growing cell experiments, the cells were loaded onto the chip in stationary phase as described previously. For the experiments on growing cells, chips containing stationary phase *E. coli* were then flushed with a continuous flow of fresh LB (100 μl h^–1^) for 3 h using the 5 ml syringe, which led the cells to start growing and dividing. At the end of this 3 h growth period, the LB syringe was disconnected from the chip and replaced with a 100 μl syringe containing minimal media with 1 g L^–1^ glucose, and the chip was flushed for 10 min (at 300 μl h^–1^) with this solution to wash away the LB. The glucose was added to the minimal media to prevent the cells from starving. Thereafter, the syringes were again exchanged and the ofloxacin solution (100 × MIC, 12.5 μg ml^–1^ dissolved in PBS, 250 μl syringe) was perfused through the chip at 100 μl h^–1^, with fluorescence images acquired at 5 s intervals. The use of microfluidic flows and fluorescence imaging enabled us to precisely track the arrival of the drug in the vicinity of the cells, with cellular drug accumulation imaged simultaneously with drug dosage. It must be noted that to reduce the background auto-fluorescence at 365 nm, prior to the ofloxacin flush the imaging area was bleached with the excitation light for 5 s. As detailed below, we performed controls with propidium iodide staining after UV and ofloxacin exposure to confirm that the UV light used did not compromise the cells' membrane integrity.

For experiments on stationary phase cells, the chips containing stationary phase *E. coli* were flushed for 10 min with PBS (300 μl h^–1^) to wash away residual LB, the imaging area was bleached for 5 s with the UV light (365 nm) and subsequently the ofloxacin was perfused through the chip, with the drug concentration and imaging settings exactly the same as for the growing cell experiments.

For both growing and stationary phase cell experiments, we performed auto-fluorescence controls (ESI† Notes 2 and 3) where instead of the ofloxacin, PBS was perfused through the chip (the rest of the protocols remained identical). A representative dataset is reported in Fig. S1 in the ESI;† the complete datasets of all the auto-fluorescence controls performed are provided as supplemental data.

### Image analysis

The image analysis was performed using a custom Python module.^24^ First, a specified range of frames of the dataset are loaded. Optionally, manually selected out-of-focus time-points are ignored. Cell detection is performed on a frame-by-frame basis as follows. First the frame is filtered using a difference-of-Gaussian (DoG) scale-space filter^25^ spanning a small range of scales, corresponding to the scale range of bacterial widths. The resulting scale-space volume is maximum-projected along the scale axis, and the automatic threshold detected using the Triangle method.^26^

The centroids of the regions in the binary image resulting from applying this threshold are used to determine the axis of the side channels by using principal component analysis. The axis of the side channels is then used to determine the upper and lower extents of the side-channel-region, which are then used to generate a side-channel-region mask, in addition to two candidate main-channel-region masks. The side-channel-region mask is then used to select bacterial regions from the binary image. The correct channel is identified from the two candidate regions by analysing the fluorescence for the region whose mean signal exhibits the most variation.

Cells are tracked frame-to-frame by matching positions such that nearest-matching bacteria are assigned only if the match is cross-validated in both forward and backward temporal directions.^27^ Bacterial trajectories are filtered to remove short trajectories (less than 10% of the full length).

The final trajectories are analysed as follows. First, a pre-determined dark-count (which is the average intensity of an image captured with the camera sensor covered) is subtracted from each bacterium's mean fluorescence, yielding the dark-count-corrected mean intensities. The corresponding dark-count-corrected PDMS background values for each bacterium are obtained by averaging the pixel intensity values of the PDMS to the immediate left and right of the individual bacterium and applying a similar dark-count correction. This bacterium-specific dark-count-corrected PDMS background is subtracted from the corresponding bacterium. Finally, the background subtracted bacterium's intensity at the starting time point is subtracted from all the values at later time points, yielding the background corrected bacterial fluorescence profiles over the course of the experiment.

For the drug dosage fluorescence, the initial intensity value of the dosage “main” channel (dark-count-corrected) is subtracted from all subsequent time points to initialise the drug fluorescence value to 0 (before drug arrival) – this also accounts for the subtraction of the background in the main channel. This reveals the drug dosage fluorescence profile over the course of the experiment.

To account for any differences in absolute drug fluorescence between experiments, for the comparative analysis of drug accumulation across the different experiments, all the background corrected cell and drug dosage fluorescence values in an experiment are normalized to the final value of the drug fluorescence in the main channel (*t* = 400 s) for that experiment. Note that this drug fluorescence value at *t* = 400 s is post-subtraction of the initial main channel background (measured before drug arrival) and thus always corresponds to the same concentration of ofloxacin (100 × MIC, 12.5 μg ml^–1^) across all experiments. These values are used for all comparative analysis and modelling results in the paper. It is important to note that, since we are using this normalization in the model, we are assuming that the correspondence between drug fluorescence and concentration is the same in the main channel and in the vicinity of the cells. It is not possible to accurately resolve the drug fluorescence in the side channels in the immediate vicinity of each cell. The cells themselves are brighter than the surrounding channel and are hence easier to detect and track and, as specified above, we have established a protocol to subtract the scattering and fluorescence background for the cells.

Finally, since the cellular auto-fluorescence temporal profiles were flat (Fig. S1B and D†), we did not need to correct for this effect when analysing the drug accumulation experimental data; we simply subtracted the initial cellular fluorescence (at *t* = 0) from the cell fluorescence at all the time-points, as detailed above. We should also mention that the automated tracking works better for growing cells than for stationary phase cells, which were smaller in size and therefore more difficult to detect. However, this does not significantly affect the average results (Fig. S2†), and the cell fluorescence values obtained through the automated code were similar to those obtained by manually selecting and measuring the cells in ImageJ; since we do not fit the model to the data for stationary phase cells, we used the automated tracking results in all the figures in this manuscript. Additionally, we manually checked the data post the automated image analysis to discard certain objects that were incorrectly detected.

### Propidium iodide (PI) staining to test membrane integrity after UV and ofloxacin treatment

To ensure that the combination of UV (365 nm) exposure and ofloxacin treatment does not compromise the cells' membranes, we treated WT *E. coli* cells (growing) after an experiment with PI (1 μl dissolved in 1 ml PBS) for 10 min at a flow rate of 100 μl h^–1^. PI is a stain commonly used to identify bacterial cells with compromised membranes. PI fluorescence was captured using an mCherry filter set (Chroma) using the green LED for excitation. A combined bright-field and mCherry fluorescence image representative of these experiments is shown in Fig. S3,† where it can be seen that less than 5% of the cells are stained with PI. Similar levels of PI staining were obtained for cells treated with ofloxacin but not bleached directly with the focused UV light. This suggests that our UV exposures do not compromise membrane integrity for the majority (>95%) of the cells.

### Mathematical model

We model drug accumulation (Fig. 4A) in the different compartments of a Gram-negative bacterium using the following set of ordinary differential equations (ODEs):i
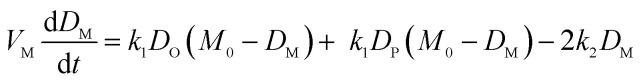

ii
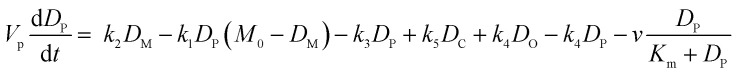

iii
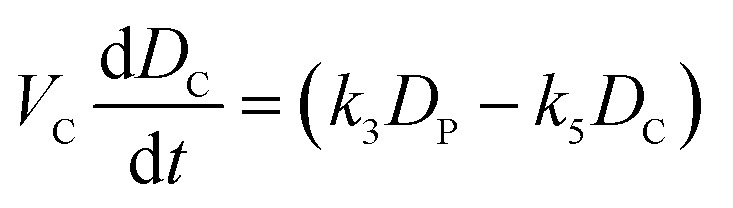
where *D*_O_, *D*_M_, *D*_P_ and *D*_C_ denote the drug concentrations in the external environment, the outer membrane, the periplasm and the cytoplasm, respectively. Importantly, we used the measured drug dosage traces for estimating *D*_O_ for every experiment, which allows us to control for any variations in the drug dosage profiles across different experiments. We model porin-mediated drug transport through the outer membrane as a two-step reversible process: drug molecules bind to porins with rate constant *k*_1_ from either side of the outer membrane and unbind to either side at rate *k*_2_. *M*_0_ denotes the concentration of functional porins in the outer membrane; based on literature values of the numbers of porins in typical Gram-negative outer membranes, we assumed that the total number of porins would vary between approximately 1 × 10^5^ to 2 × 10^5^ per cell (WT);^28^ this was used to restrict the range of possible values for *M*_0_. As a first approximation, we assume that diffusion through the LPS-lipid bilayer is negligible (*k*_4_ ∼ 0) in comparison to porin-mediated transport.^3^ Furthermore, we postulate that ofloxacin molecules, like other fluoroquinolones,^15^,^21^ diffuse across the inner membrane lipid bilayer (rate constants *k*_3_ and *k*_5_) and that the efflux of drug molecules from the periplasm to the external medium follows Michaelis–Menten kinetics with maximal rate *v* and Michaelis constant *K*_m_.^29^ Parameters *V*_M_, *V*_P_ and *V*_C_ denote the volumes of the outer membrane, periplasm and cytoplasm, respectively. The parameter *k*_3_ was calculated on the basis of passive diffusion measurements of ofloxacin permeability across lipid vesicle bilayers (Fig. S4†). To account for any potential binding of the drug to targets within the cytoplasm, we do not assume any equivalence between *k*_3_ and *k*_5_, an approach similar to that applied by Westfall *et al.*;^29^ we only make the assumption that *k*_5_ ≤ *k*_3_. Crucially, the parameters (*k*_1_, *k*_2_, *k*_5_, *M*_0_, *K*_m_, *v*) were inferred from the experimental data obtained with the WT, Δ*ompF* and Δ*tolC E. coli* strains. The total drug concentration was calculated as:iv
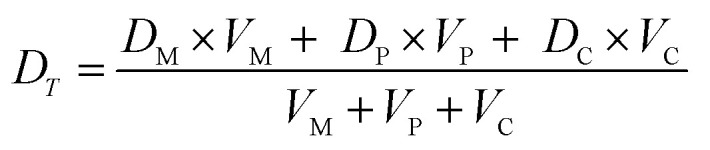
To model drug accumulation in the Δ*ompF* strain, we used equations (i)–(iii) above, additionally assuming a possible decrease in the number of porins relative to the WT, *i.e.*, *M*_0_,_Δ*ompF*_ ≤ *M*_0_. Similarly, for the case of the Δ*tolC* strain, we assumed that the *maximal* efflux rate may decrease relative to the WT, *i.e.*, *v*_Δ*tolC*_ ≤ *v*.

All model simulations were run in Matlab (R2018b) using the in-built explicit Runge–Kutta (4, 5) solver (function ode45; default settings). Details of the parameter estimations and distributions are provided in the ESI† (Fig. S5–S7).

## Results

**Fig. 1:**
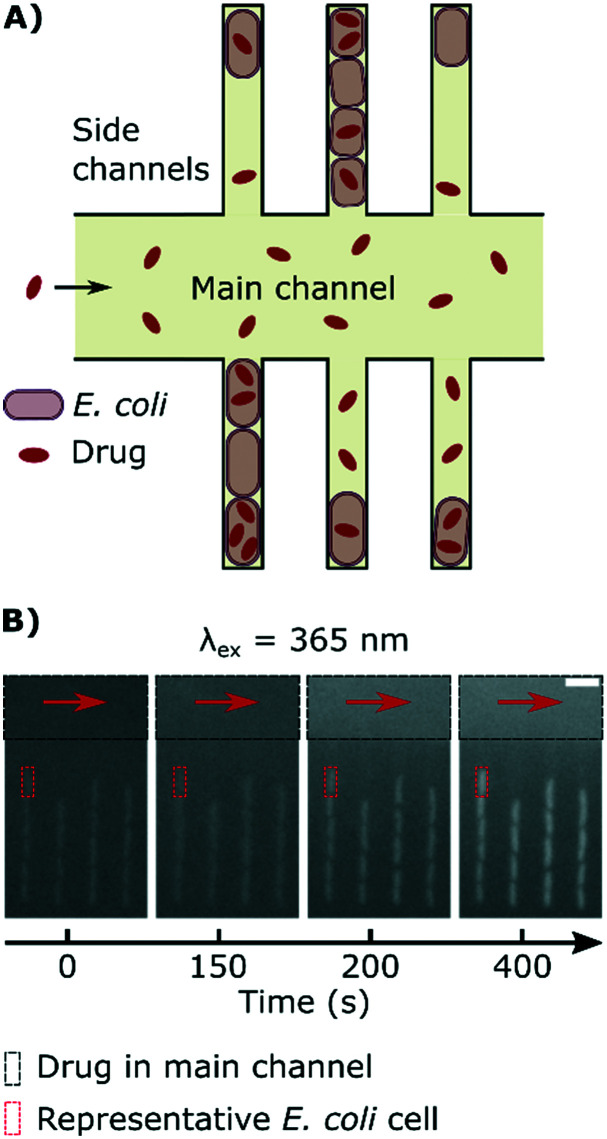
Measuring drug dosage and intracellular accumulation in individual bacteria. A) Schematic of the microfluidic chip used for the ofloxacin accumulation experiment. A main channel of height 25 μm and width 100 μm is used for continuously exchanging the microenvironment with nutrient, drug or dye delivery; cells are confined single-file in a network of side channels whose height and width are close to the cell size. B) Sequence of epifluorescence images (*λ*_ex_ = 365 nm) showing the delivery of ofloxacin (12.5 μg ml^–1^ in PBS; black rectangle) and its corresponding accumulation by the cells in the side channels; a representative individual cell is marked by a red rectangle as a guide. Scale bar = 5 μm.

Fig. 1A explains the principle behind our drug accumulation assay. Geometrically confined *E. coli* are dosed with ofloxacin, whose dosage and accumulation in the cells is tracked using its auto-fluorescence at an excitation wavelength of 365 nm. Although the cells themselves have a baseline auto-fluorescence before exposure to the drug (Fig. 1B), as noted in the Experimental section, we ran control experiments in the absence of the drug and found that the auto-fluorescence profiles remain flat during the course of the experiment (ESI† Note 2); this contribution is subtracted in our image analysis protocol.

**Fig. 2:**
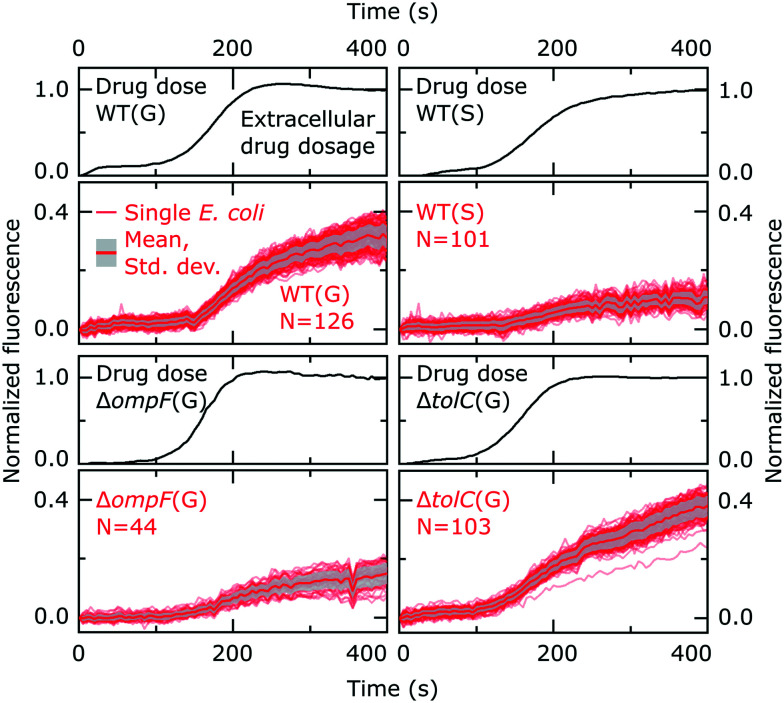
Individual ofloxacin accumulation experiments for the bacterial strains/conditions investigated. The drug dosage plots report the temporal profile of ofloxacin delivery in the separate experiments, with the subsequent plots reporting the corresponding ofloxacin accumulation profiles of the individual *E. coli* cells; the thick red line represents the mean and the grey shaded area the standard deviation of the cellular ofloxacin accumulation profiles. The cell fluorescence values are reported after correcting for the background, subtracting the initial cellular auto-fluorescence at *t* = 0, and normalizing to the fluorescence of the drug as detailed in the Experimental section. The *E. coli* strains (wild type, WT; Δ*ompF*; Δ*tolC*), conditions (growing, G; stationary phase, S) and number of cells (*N*) are indicated inset. For reference, the complete datasets (before normalization) for all strains/conditions including all the repeats are provided in Fig S8 in the ESI.†

It is important to emphasize that our approach allows us to precisely track the dosage of the drug in all our experiments, *via* its auto-fluorescence (ESI† Note 1); this allows us to study drug accumulation during drug dosage (Movie S1†), unlike the majority of population level assays where the drug is typically incubated with the bacteria for a defined period of time before the cells are analysed.^12^ Even recent single-cell approaches require bacterial pellets to first be incubated with drugs before being resuspended between coverslips for imaging,^30^ and hence do not study drug accumulation during drug dosage. In contrast, our novel assay uses microfluidics to overcome this limitation and facilitates the real-time imaging of drug accumulation in response to dosage; typical single-cell fluorescence profiles showing the accumulation of the drug in response to the dose are reported in Fig. 2. We observe an increase in cellular drug fluorescence within seconds after the arrival of the drug in the vicinity of the cells, which further illustrates the point that for drugs such as ofloxacin, accumulation studies should ideally commence as soon as the cells are dosed. We note that in the mother-machine, small molecules diffuse rapidly across the side channels, within approximately a second after arrival *via* the main channel;^14^ thus all the cells in a side channel are exposed to the same drug concentration within a second of the drug arriving *via* the main channel.

Our cellular fluorescence profiles correlate well with a previous population level study of ofloxacin accumulation in *E. coli* that involved the rapid sampling of bacteria incubated with the drug – the study showed an initial period of rapid accumulation, within 100 seconds.^31^ Ofloxacin accumulation in *E. coli* over longer timescales of up to an hour appears to be biphasic,^31^ but here we focus our attention on the initial stages of drug accumulation, in order to develop a novel assay capable of rapidly capturing the drug accumulation characteristics of individual cells.

**Fig. 3:**
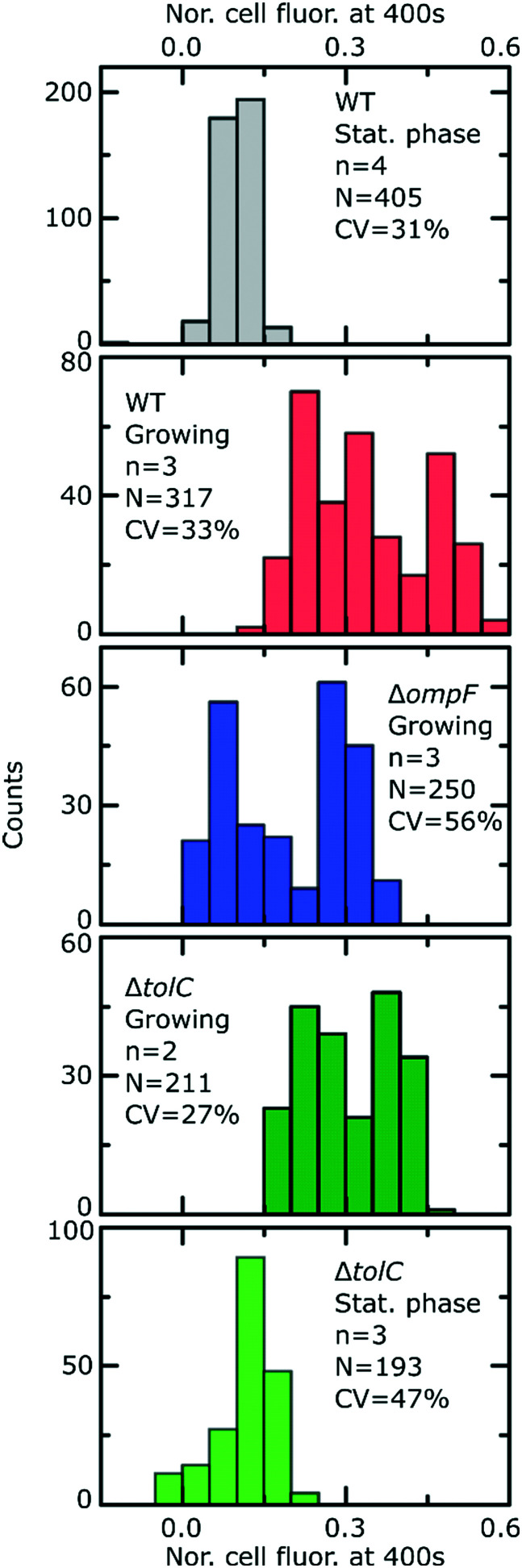
Final level of normalized whole cell fluorescence for the different strains and nutritional conditions after exposure to ofloxacin. In the insets, *n* refers to number of experimental repeats, *N* reports the total number of bacteria and CV refers to the coefficient of variation of the data across all the experiments for a given strain and condition. All comparisons are made at *t* = 400 s. To ease comparisons between the strains/conditions, a box plot summarising the data from the distributions above is provided in Fig. S9.†

Fig. 2 reports the dosage (black lines) and cellular (red lines) drug fluorescence profiles (normalized) of individual *E. coli* cells in a range of experiments. Comparing growing (G) *versus* stationary phase (S) wild type (WT) cells, we immediately observe that although the drug dosage profiles are similar in the two experiments, the cellular fluorescence (and hence drug accumulation) profiles are very different for stationary phase *versus* growing cells. We measured significantly lower intracellular drug fluorescence levels in stationary phase cells compared to cells that were growing; this also shows that our microfluidic technique is capable of rapidly distinguishing between cells that readily accumulate ofloxacin *versus* those that show a dramatically reduced accumulation profile. To quantify this difference, we compared the distributions of cellular fluorescence (normalized to the value of drug dosage fluorescence) at *t* = 400 s across all experimental repeats in Fig. 3. Over all datasets, growing WT cells show an approximately 3-fold higher fluorescence than stationary phase cells (Table 1; *p* < 10^–10^; 2 sample *t*-test with Welch's correction). A similar result was obtained when comparing growing and stationary phase cells in the Δ*tolC* mutant strain (Table 1; *p* < 10^–10^). These differences also suggest that our fluorescence assay is not limited by any non-specific binding of ofloxacin to outer membrane components, which should be of a similar magnitude for stationary phase and growing cells.

**Table 1:** Summary of the results of the normalized fluorescence values of cells from the different *E. coli* strains investigated after treatment with ofloxacin (at *t* = 400 s)

*E. coli* strain	Growth phase	Number of cells	Normalized fluorescence at *t* = 400 s after ofloxacin treatment (mean ± s.d.)
Wild type	Growing	317	0.34 ± 0.11
Wild type	Stationary phase	405	0.10 ± 0.03
Δ*ompF*	Growing	250	0.20 ± 0.11
Δ*tolC*	Growing	211	0.31 ± 0.08
Δ*tolC*	Stationary phase	193	0.12 ± 0.06

From Fig. 2, we also observe, within a measurement timeframe of just 400 s, that the growing Δ*ompF* mutant strain accumulates lower amounts of ofloxacin than the WT (growing) (Table 1; *p* < 10^–10^). Thus the absence of the OmpF porin lowers the ability of ofloxacin to permeate into the cell compared to the WT strain. Our result agrees with previous reports that show that OmpF facilitates fluoroquinolone transport across Gram-negative outer membranes.^4^,^32^

Interestingly, we were unable to detect an increase in ofloxacin accumulation in growing Δ*tolC* mutant cells compared to the WT at the 400 s time-point (Fig. 3). In fact, we measured a small decrease in the drug fluorescence in growing Δ*tolC* cells compared to the growing WT cells (Table 1; *p* = 2.7 × 10^–4^).

Importantly, our ability to directly compare drug accumulation in different metabolic states revealed that the growing Δ*ompF* mutant strain accumulates more ofloxacin than the stationary phase WT (Table 1; *p* < 10^–10^), suggesting that the growth phase plays an even bigger role in ofloxacin accumulation than the complete removal of a major pathway for drug accumulation that has often been linked to antibiotic resistance.^33^ We believe this is the first time such a direct comparison has been performed. These results emphasize the importance of studying the role of the cellular metabolic state in drug accumulation.

**Fig. 4:**
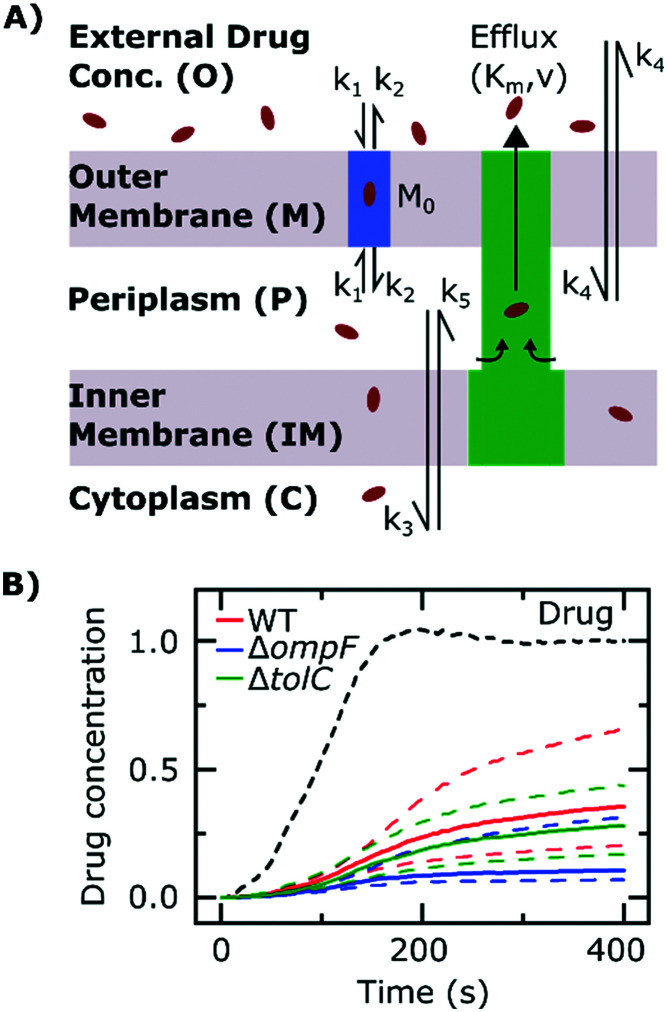
Mathematical modelling of the drug transport process. A) Schematic of the main processes involved in drug translocation across Gram-negative cell envelopes. Drug molecules penetrate the outer membrane (*M*) primarily through protein porins, with association and dissociation rates *k*_1_ and *k*_2_, respectively. *M*_0_ refers to the concentration of functional porin binding sites in the outer membrane. Any residual (non-porin) transport across the outer membrane LPS barrier is modelled with *k*_4_. Drug transport through the inner membrane is modelled with kinetic parameters *k*_3_ and *k*_5_. Drug molecules are subject to removal from the cell *via* active efflux mechanisms which follow Michaelis–Menten kinetics (*K*_m_, *v*). B) The temporal dependence of the normalized drug concentration in cells for WT (red), Δ*ompF* (blue) and Δ*tolC* (green) bacteria in response to the drug dosage input (dashed black line) is in line with the experimental results on growing cells. Note that the “concentrations” here include both free and bound drug molecules. These drug accumulation profiles were obtained by using the kinetic parameter values in Fig. S5A and B† and the theoretical model (eqn (i)–(iii)). The concentrations reported are normalized to the drug dosage concentration. The solid lines correspond to median accumulation in the cells and the dashed lines represent the [20,80] posterior predictive intervals. The results shown were generated by running the model using 500 independent samples of parameters 
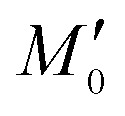
 and *v*′ from their joint posterior distributions (details in ESI†). All other parameters were fixed to the values given in Table S2.†

To further probe the transport processes underlying ofloxacin accumulation in individual cells, we complemented our experimental results with mathematical modelling, depicted schematically in Fig. 4A (the model equations were presented earlier along with the experimental methods). The use of mathematical modelling and Bayesian inference to rationalize our data enabled us to maximize the information embedded in our time-lapse single-cell measurements, leading to predictions of the dynamics of the accumulation process.

We extracted kinetic parameters corresponding to the single-cell drug accumulation profiles and quantified changes in these parameters in the different strains (Fig. S5A and B†). To validate our inference procedure, we used data simulated by the model and showed that we can indeed recover the parameter values which were used for generating these (Fig. S10†). The modelled drug accumulation profiles obtained from these kinetic parameters recapitulated the experimental drug accumulation trends we observed at the whole-cell level (Fig. 4B). Note that in all our discussions of accumulation and concentrations, we refer to “concentrations” simply as the total number of molecules in a compartment (cellular or subcellular) divided by the volume of the compartment – hence free and bound molecules are both counted as contributing towards the “concentration” and “accumulation” in this context.

The model also allowed us to predict drug accumulation in the different subcellular compartments (Fig. S5C†), which is a major challenge for the entire research community working in the field of antibiotic drug development (the detailed predictions are described in the ESI†). Briefly, the model predicts that the periplasm contains approximately 30-fold lower ofloxacin concentrations than the cytoplasm for all three strains at *t* = 400 s – this is likely due to the binding of the ofloxacin molecules to targets within the cytoplasm. The model also predicts a lag time of approximately 100 s between drug accumulation in the outer membrane *versus* drug accumulation in the cytoplasm. It is important to note that these are *predictions*, arising out of our whole-cell data; validation of the model predictions regarding subcellular levels of drug concentration will only be possible once the considerable experimental challenges for these measurements are overcome. Future work will also involve studying drug accumulation after modulation of other transport pathways in the Gram-negative double membrane to estimate their relative contributions to drug accumulation at the subcellular level.

## Discussion

Antibiotic accumulation in Gram-negative bacteria is an extremely complex biophysical phenomenon because of the different physicochemical pathways and combination of active and passive transport processes involved. It is essential to understand the roles of these pathways in a quantitative manner to rationally design drugs that can accumulate in the vicinity of their targets, which will crucially contribute to overcoming the void in Gram-negative drug discovery. This has proved exceptionally challenging, in part due to the lack of investigative techniques that can study these processes in well-controlled environments, with single-cell resolution.

We have leveraged the power of microfluidics to develop a rapid, single-cell assay to quantify antibiotic accumulation in Gram-negative bacteria. We demonstrated that we can distinguish between cells that show dramatically different drug accumulation profiles within measurement timescales of under 400 s. This complements the recent drive towards rapid antibiotic susceptibility tests that can inform on the susceptibility of pathogenic bacteria to antibiotics within 30 minutes, as opposed to traditional minimum inhibitory concentration studies that typically take hours to days to provide results;^20^ such rapid testing is critically important in clinical settings. Unlike the majority of techniques, which involve complex washing steps after drug delivery, or are limited to certain specific media conditions,^3^,^10^ our microfluidic platform facilitates the study of drug accumulation in different microenvironments and cellular metabolic states. We quantify drug dosage in every experiment, which allows us to correct for any variations in fluorescence intensities/flow conditions between experiments. Since we use microfluidics, we quantify drug accumulation from the moment the drug arrives in the vicinity of the cells, facilitating the real-time measurement of the transport process.

Furthermore, since our excitation wavelength is 365 nm (ESI† Note 4), in contrast to previous studies using deep UV illumination to study antibiotic accumulation in single cells,^11^,^34^ we can work with standard optics and light sources, rather than needing quartz objectives and cover slips, and deep UV light sources which may not be easily accessible. This will facilitate the use of our assay in non-specialized laboratories.

Using our novel approach, we established that within the timescales investigated, ofloxacin accumulates to a greater degree in growing *versus* stationary phase bacteria (Fig. 2 and 3). It is likely that this reduction in ofloxacin accumulation contributes to the significant increase in cell survival to this drug that was previously observed as the cells enter stationary phase compared with early exponential phase cultures,^35^,^36^ since ofloxacin's antibacterial activity depends on its accumulation in the cytoplasm of the bacteria. It is interesting to speculate on whether this difference in accumulation is mediated by differences in the expression of the target or whether it is due to differences in the expression of the drug transport pathways. Fluoroquinolone antibiotics target DNA gyrase and particularly its complexes with DNA,^37^,^38^ which regulate DNA supercoiling. The levels of the Gyr proteins themselves do not change appreciably as cells grow from exponential into stationary phase, even after 72 h of starvation.^39^ However, quantifying the levels of DNA gyrase–DNA complexes is more difficult; the fact that stationary phase cells display a distribution of relaxed (as opposed to supercoiled) plasmids suggests that the levels of DNA gyrase–DNA complexes decrease as cells progress into stationary phase,^39^ which could explain the differences in drug accumulation between the states. We note that these DNA gyrase–DNA complexes (and potentially other intracellular components) act as a sink for the ofloxacin molecules once they enter the cell, which explains the higher fluorescence of the cells compared to the background observed post drug exposure in Fig. 1B.

On the other hand, transcriptomic studies have also revealed that the expression of the genes encoding the major *E. coli* porins OmpF and LamB, through which antibiotics diffuse, was significantly upregulated in exponentially growing compared to stationary phase *E. coli* cells (Fig. S11† (ref. 35)). This suggests that the differences in ofloxacin accumulation that we observe between growing and stationary phase cells may also be due to the differential expression of key membrane transport pathways. However, this will require further investigation. Future studies on the differences in accumulation between cells in different physiological states may also contribute towards a better understanding of antibiotic tolerance; indeed, it is known that growth conditions play an important role in the development of tolerance to various antibiotic classes.^40^ The technique presented in this paper offers a platform for the investigation of these observations in the context of differential drug and metabolite uptake,^55^–^60^ complementing efforts to characterize phenotypic heterogeneity such as antibiotic tolerance and persistence.^9^,^41^–^44^

Using biophysical experimental model systems, we and others have previously shown *in vitro* that porins such as OmpF facilitate fluoroquinolone transport across the outer membrane,^5^,^32^ and that fluoroquinolones also diffuse freely across phospholipid bilayers such as those found in the cytoplasmic membrane.^15^ In our experiments on growing cells, knocking out the *ompF* gene led to a decrease in drug accumulation compared to the WT strain, confirming that fluoroquinolones utilize porins to enter *E. coli* cells. However, the effect of the growth phase was more significant than the removal of this porin – stationary phase WT cells accumulated significantly lower amounts of ofloxacin than the growing Δ*ompF* mutant (Fig. 3, S9†). Previous studies have reported that nutrient-starved bacteria show reduced drug accumulation,^45^ but these studies did not determine the extent to which environmental factors, and subsequent cell phenotypic acclimation, predetermine drug accumulation compared to genotypic changes which result in the loss of membrane transport pathways.

On the other hand, the role of the TolC efflux protein in fluoroquinolone transport is currently a matter of debate. As described in the Results, we did not measure any increase in drug accumulation in the Δ*tolC* strain, compared to the WT strain (growing cells). The TolC outer membrane efflux protein forms an important part of multi-drug efflux systems such as AcrAB-TolC that eject antibiotics and other toxins from *E. coli* cells.^46^ Therefore, naively one would have expected that losing TolC negatively affects the ability of the cell to efflux the antibiotic, thus increasing its intracellular accumulation. However, cellular fluoroquinolone accumulation data comparing parental WT strains and their corresponding *tolC* knockouts show contradictions, with some reports showing increased accumulation^47^ in the knockout and others showing no significant differences between the strains.^48^ Further, although the overproduction of the AcrAB-TolC efflux system has been implicated in the antibiotic resistance of clinical isolates of *E. coli* species, there was no significant correlation between the overexpression of the *acrAB* and *tolC* genes.^46^,^49^ With regards to fluoroquinolone antibiotics, it was reported that average *tolC* expression levels in fluoroquinolone-susceptible and fluoroquinolone-resistant clinical isolates of *E. coli* were not statistically different.^46^,^49^ Zgurskaya and co-workers therefore concluded that TolC quantities alone do not limit the drug efflux capabilities of *E. coli*.^46^ Our data further corroborate this hypothesis.

In terms of future developments (ESI† Note 5), our microfluidic approach offers possibilities for scaling up the number of drugs/pathogens that can be tested on the same chip, *via* parallelization of the cell trapping chambers. On the drug development side, the assay also has the advantage of needing only micrograms of chemicals for testing, which is important when evaluating novel, candidate drugs that are typically expensive to manufacture. Our readout is based on fluorescence, and can be used to test the cell permeation properties of new fluorescent antibiotic probes that have been developed for a range of antibiotics including penicillins, glycopeptides, polymixins, oxazolidinones, trimethoprim, macrolides and fluoroquinolones;^50^–^54^ similar probes can be developed for newly discovered antimicrobials as well. The use of more sensitive fluorescent probes could also let us potentially correlate cell survival to reduced accumulation in longer-term experiments in the future. Our assay could also be used to study the influence of specific functional groups on the cellular accumulation of closely related compounds. For instance, biophysical *in vitro* measurements of different fluoroquinolones revealed orders of magnitude differences in their lipid permeabilities;^15^ our system facilitates similar studies on live bacteria themselves.

## Conclusions

We have developed a novel approach to investigate antibiotic accumulation in individual Gram-negative bacteria in well-controlled microenvironments. Our experiments enabled us to quantify the role of the nutrient microenvironment and metabolic state of the cells in drug accumulation at the single-cell level. We reported, to the best of our knowledge, the first quantitative comparisons between drug accumulation in cells in different metabolic states and in cells with specific transport pathways disabled. Combining our data with mathematical modelling and Bayesian inference enabled us to predict the kinetic parameters underlying ofloxacin accumulation in individual *E. coli* cells. As discussed, our technology represents a step-change in the way drug accumulation in bacteria is studied, with potential benefits to the drug development and diagnostics industries, infectious disease specialists, as well as to the large academic community studying antibiotic transport in Gram-negative organisms. The experimental setup is relatively simple to implement on standard epi-fluorescence microscopes and will provide researchers with a new, transferrable platform with which to study this vitally important permeation process in a range of pathogenic microbes.

## Conflicts of interest

There are no conflicts to declare.

## Data availability

All the data is available in the main text or in the ESI.† The codes for the modelling and the image analysis are available *via* online repositories. Please contact the corresponding authors for access to the codes.

## Supplementary Material

Supplementary information

Supplementary information

Supplementary movie
