# Supplementary material for: Single-cell microfluidics facilitates the rapid quantification of antibiotic accumulation in Gram-negative bacteria
Source: Lab Chip. 2020 Jun 16;20(15):2765–75. doi: 10.1039/d0lc00242a (PMC7953842; doi:10.1039/d0lc00242a)
Supplement: Supplementary file 1 [file LC-020-D0LC00242A-s001.pdf]

Electronic Supplementary Material (ESI) for Lab on a Chip.
This journal is © The Royal Society of Chemistry 2020

Supplementary Materials for

Single-cell microfluidics facilitates the rapid quantification of antibiotic accumulation in Gram-negative bacteria

Authors: Jehangir Cama[a,b,c], Margaritis Voliotis[a,b], Jeremy Metz[a,d], Ashley Smith[a,d], Jari Iannucci[a,d], Ulrich F. Keyser[c], Krasimira Tsaneva-Atanasova[a,b] and Stefano Pagliara[a,d].

a. Living Systems Institute, University of Exeter, Exeter EX4 4QD, United Kingdom.

b. College of Engineering, Mathematics and Physical Sciences, University of Exeter, Exeter EX4 4QF, United Kingdom.

c. Cavendish Laboratory, Department of Physics, University of Cambridge, JJ Thomson Avenue, Cambridge CB3 0HE, United Kingdom.

d. School of Biosciences, College of Life and Environmental Sciences, University of Exeter, Exeter EX4 4QD, United Kingdom

Correspondence to: j.cama@exeter.ac.uk, S.Pagliara@exeter.ac.uk

This PDF file includes:

Supplemental Notes 1-5
Details of the mathematical model and its predictions
Supplementary references
Figures S1 to S11
Tables S1 to S4
Caption for Movie S1

Other Supplementary Materials for this manuscript include the following:

Movie S1
Datasets for auto-fluorescence control experiments

Supplemental Note 1: Quantifying drug dosage

Our experimental approach, using syringe pumps and microfluidic devices to control drug delivery and auto-fluorescence for drug detection (Experimental section), enables us to precisely quantify the arrival of the drug in the vicinity of the cells under investigation. Representative images of the main, drug delivery channel and the side, cell-hosting channels before and after drug dosage are shown in Figure 1B (main text). Example drug dosage profiles are reported in Figure 2 of the main text and in this document (dashed black lines in Figures S1A,C and S8). Since the drug dosage concentration is the same across all experiments, this measurement allows us to correct for any drug fluorescence intensity variation across all the different experiments. We measure the background at $t = 0$, and observe that the drug arrives in the field of view typically around 100 s after the start of the experiment, reaching its final concentration around 200 s (Figure 2). We use the final, steady-state value of the drug dose fluorescence (at $t = 400$ s) to map drug fluorescence to drug concentration (see Experimental section). Importantly, since we measure the drug dosage profile across different experiments, we use this information as an input to the model, which allows us to account for any differences between the dose profiles across the different experiments.

Supplemental Note 2: Quantifying cellular auto-fluorescence

For each strain/condition, we performed control experiments to measure the auto-fluorescence profiles of individual bacteria in the absence of the drug (see Experimental section). A representative comparison between cellular drug fluorescence and auto-fluorescence profiles is reported in Figure S1, corresponding to either the presence (Figure S1A,C) or absence (Figure S1B,D) of the drug. We observe that the cellular control auto-fluorescence profiles are flat across the timescales of the experiment; thus cellular auto-fluorescence has a negligible effect on the drug accumulation profiles. Similar cellular auto-fluorescence profiles were observed across all the control experiments performed (number of experimental repeats: 2 (WT growing), 3 (Δ*ompF* growing), 2 (Δ*tolC* growing), 3 (WT stationary phase) and 3 (Δ*tolC* stationary phase) – datasets uploaded as supplemental material).

Supplemental Note 3: Quantifying intra-experimental variability

In order to estimate the variation in cellular fluorescence in the absence of the drug, we used the auto-fluorescence control experiment shown in Figure S1B to estimate the underlying biological and systematic variation in our experiments. These measurements report the auto-fluorescence of the same cells measured at different time points in the experiment. We quantified the coefficient of variation (CV) of the cell auto-fluorescence intensities (over the timescales of the experiment) of the 103 individual cells shown in Figure S1B. The mean CV across all the cells was 10 ± 3 % ($N = 103$, mean ± s.d.). This gives a quantitative estimate of the measurement (systematic and underlying biological) heterogeneity for individual cells within a single experiment.

We compare this variability in cellular auto-fluorescence with the apparent heterogeneity in drug accumulation in the cells in Figure S1A. To estimate this value, we measured the intensity of the cells at the end of the drug accumulation experiment ($t = 400$ s). The heterogeneity in the cellular fluorescence corresponding to drug accumulation (in the knowledge that this includes the systematic and underlying biological variation mentioned above) is extracted by measuring the CV of the fluorescence across all the cells at this time-point. *Unlike* the CV measurement of the control which was for *individual cells* across *all* time-points, to estimate drug accumulation heterogeneity amongst the 126 different cells, we measured the CV in the fluorescence of *all the cells* at the *final* time-point. This analysis yields a CV of 9.7%, similar to the CV in the auto-fluorescence intensities of untreated cells mentioned above.

Supplemental Note 4: Metabolite auto-fluorescence at 365 nm

Although cellular metabolites may also fluoresce at similar wavelengths to our excitation wavelength (365 nm), we have corrected this by subtracting the baseline cellular fluorescence as described in the Experimental section (and in Supplemental Note 2). We note that metabolite concentrations are known to fluctuate in response to fluoroquinolone treatment, but this is typically less than a two-fold change within the timescales of our experiment and includes both increases and decreases[1]. The baseline cellular auto-fluorescence (growing WT cells, Figure S1B) shows typical intensities of approximately 1700 (arb. units), while the fluorescence *increases* in the cells due to drug accumulation are approximately 5200 (arb. units, Figure S1C). Therefore, we estimate that the *maximum* contribution of metabolites to our fluorescence signal, in the case where *all* the metabolites were to double in number (and assuming that the fluorescence scales linearly), would be approximately 33% in this case; however, considering that the metabolites show both increases and decreases in response to fluoroquinolone treatment, we estimate that the actual contribution is significantly lower, and would constitute a higher order correction to our results. Note that to the best of our knowledge, a non-fluorescent version of ofloxacin does not exist, making a direct measurement of the changes in metabolite auto-fluorescence in response to ofloxacin treatment intractable. However, we reiterate that the baseline cellular auto-fluorescence is already accounted for in our analysis.

Studies on the fluorescence of the ofloxacin molecule itself in mixed aqueous-organic solvents (used to mimic biological systems) also show that the fluorescence quantum yield of ofloxacin is relatively stable to changes in the solvent composition, as long as the percentage of water (by weight) in the mixture is above approximately 12%[2]. *E. coli* cells are approximately 70% water (by weight)[3], and hence the fluorescence yield of ofloxacin once inside the cells should not be much different (due to changes in the cytosolic "solvent") in growing versus stationary phase cells. We also note that although the fluorescence of ofloxacin is affected by pH, the yield is relatively stable to pH changes around physiological pH values[4]. A study on ofloxacin showed that when excited at 340 nm (near the excitation wavelength of 365 nm used in the present study), the fluorescence yield remained steady between pH values of 3 and 7 and then "dropped gradually"[4]. Furthermore, the intracellular pH of *E. coli* BW25113 remains steady around 7.3 through the growth cycle in LB[5]; this is only slightly lower than the pH of the drug when dissolved in PBS (approximately 7.5-7.6). In light of the above considerations, we do not expect any significant pH mediated changes in the fluorescence properties of ofloxacin when it is present in cells that are growing versus stationary phase cells.

Supplemental Note 5: Suggestions for future improvements and outlooks for the microfluidic assay

In the interests of facilitating the future development and widespread use of our methodology, we discuss ways by which the technique could be improved, based on what we learnt through our work and with the resources available to us at the time we performed the experiments.

Choice of media: Our choice of LB as the growth media was guided by the fact that we had previously profiled the entire transcriptome of *E. coli* BW25113 cells growing in LB[6]. This enabled us to correlate our drug accumulation results with, for example, changes in porin expression as shown in Figure S11 below. However, LB itself is strongly fluorescent (at 365 nm excitation) and we therefore needed to wash away the background LB with an M9-glucose solution prior to the ofloxacin challenge (note this refers to the LB in the channel itself; it is likely that some LB is also absorbed by the PDMS, which typically leads to a higher background fluorescence in the growing cell experiments compared to the stationary phase experiments, which we accounted for in the image analysis). It is also possible that some residual LB still flows in with the drug initially, which may explain some of the finer features of our drug dosage

fluorescence curves in the growing cell experiments (for example, we sometimes see a slight decrease from the peak fluorescence before the final intensity plateau). LB is also a complex media. It would be easier for future users to grow their cells in more defined culture media, particularly those that do not have strong intrinsic fluorescence. They may also then be able to study drug uptake in their growth media itself, rather than in PBS. However, we strongly urge future users who are comparing drug accumulation in different cellular growth states to ensure that the pH of the drug solution is the same in all their comparative measurements, since pH often affects the charge state of antibiotics and hence affects their membrane transport characteristics[7].

Syringe vs Pressure driven pumps: We used syringe pumps to control our flows. Syringe pumps are widely available, and some models are relatively cost-effective, which we believe will help with the wide uptake of our assay. However, the disadvantage of using syringe pumps is that each time the solutions are exchanged, the chip is disturbed since the tubing needs to be removed and then re-inserted with the new syringe connection (we believe it is important to use separate syringes for separate solutions to prevent any cross contamination). The alternative is to use pressure driven pumps, but importantly with accessories that enable the control of flow rate rather than just the pressure. Such systems are available from companies such as Fluigent (we now increasingly use their pressure driven pumps and flow control units for mother-machine experiments). One of the advantages gained is that all fluid exchanges are effected simply by replacing fluid filled vials off chip, rather than changing any tubing connected to the chip itself. This also reduces variability in the flow profiles, since the risk of damage to the inlet port by multiple cycles of removing and re-inserting tubing is reduced – the tubing only needs to be inserted once. This also minimizes the risks of debris building up near the inlet, which may also cause pulsations or irregularities in the flow. The tubing typically used with the Fluigent system is also thinner than the tubing we used with our syringe pumps. This leads to a 4-fold reduction in the volume of the inlet/outlet columns; the biopsy punch we use for the Fluigent tubing is 0.75 mm in diameter, as opposed to the 1.5 mm diameter punch used for the devices in this paper. However, using a pressure-based system may increase the cost per inlet compared to standard syringe pumps. We reiterate that even with syringe pumps, the important advantage of using our technique is that the drug dosage is measured in every experiment, and hence can be corrected for (as we have done in our model, where we use the drug dosage profile as an input). It is also worth noting that all such drug accumulation studies will likely involve slight variations in drug dose between experiments, but most techniques do not measure and account for such variations.

Choice of organism: We performed our studies with the rod shaped bacterium *E. coli*, but the side channels of the mother-machine device can also be used to trap bacteria with different shapes. For example, we have successfully trapped the spherical bacterium *Staphylococcus aureus* (unpublished) for studies in the same device. The advantage of microfluidics is that the devices can be custom designed to optimize the trapping efficiency of cells of various shapes and sizes, and we envisage that future such drug accumulation studies will be performed on a range of clinically relevant microorganisms of diverse shapes and sizes.

Outlooks: Besides the suggestions provided in the sections above, we would like to mention that we chose a short experimental timescale to determine whether we could distinguish between drug accumulation in different strains/conditions within a relatively short period of time (under 10 minutes). However, there is nothing to prevent drug accumulation and/or survival studies over longer timescales – this is particularly relevant, for instance, if one wished to correlate reduced drug accumulation with cell survival. Such studies would involve measuring drug accumulation at the single-cell level, and then flushing in fresh nutrients to study the regrowth of any survivors. This is of particular relevance to studies of antibiotic tolerance and persistence[8]. It should be noted that studies on persistence and other rare

phenotypes are greatly facilitated by the use of single-cell assays – population level assays on trillions of cells often lack the resolution to identify the full range of diverse, naturally occurring phenotypes that are crucial for understanding the detailed characteristics of a community of single-celled organisms[9]. Our technique allows versatility in the duration of various stages of the assay. The microfluidics could also be parallelized, enabling the same culture to be studied at various physiological states – one could trap cells in parallel mother-machines, grow them into different metabolic states and then sequentially test drug accumulation (or, alternatively, test different drugs in parallel on the same isogenic population of cells in the same metabolic state).

Furthermore, we have used (auto)fluorescence microscopy to detect and track ofloxacin; as discussed in the main text, a range of antibiotics are indeed intrinsically fluorescent. Besides epi-fluorescence microscopy, future users may wish to explore the use of more advanced fluorescence imaging techniques with these molecules. We note that our experimental study did not investigate drug fluorescence in cellular subsections; we used mathematical modelling to infer subcellular drug concentrations. If future experiments involve localizing the fluorescence signal of the drug to certain regions of the cell, and if such regions are on the order of single pixels in size with the detectors used, the researchers should consider deconvolving their images with the point spread function of their microscope. However, this is not essential when working with relatively uniform intensity structures that are much larger than single pixels, and is also not essential when working with *relative* intensity measurements (both these conditions apply to the measurements in this paper)[10].

It is also worth noting that the microfluidics is decoupled from the imaging modality – future developments may involve the use of other label-free optical techniques such as Raman microscopy or absorbance microscopy/spectroscopy for tracking the drug molecules, which would enable an even larger set of drug compounds to be investigated. The important advantage of using microfluidics, besides the single-cell level analysis, is that it enables the investigation of cells in defined metabolic states and microenvironments, which, as we have demonstrated, may play a significant role in drug accumulation. We envisage that this property of the assay will be combined with a range of different detection modalities in the future.

Model parameter estimation:

We obtained priors for our Bayesian parameter estimation by computing maximum likelihood estimates (MLEs) of the free model parameters (Table S2) using the medians of the drug accumulation profiles for all the cells in an experiment. Please note that for convenience we use the term “population-averaged” throughout the text to refer to these *median* values of the drug accumulation profiles. Since our data was normalized based on the fluorescence of the drug dose (see Experimental section; image analysis), estimates of parameters k_1, M_0, K_m, v incorporate a constant factor related to the concentration of the drug dose (see Table S2). We denote the scaled version of these parameters using the prime symbol ($'$). We compiled a library of 18 datasets by considering all the permutations of population-averaged profiles from: (i) growing WT cells (3 experimental repeats); (ii) growing Δ*ompF* cells (3 experimental repeats) and (iii) growing Δ*tolC* cells (2 experimental repeats). We obtained parameter MLEs from each dataset, and to mitigate the risk of overfitting we then selected out of those parameter vectors the one that best fitted all 18 datasets. Under the assumption of Gaussian measurement error, the MLEs for each dataset correspond to parameter values minimising the following sum of squares: $\epsilon = \sum_t \frac{(D_{T,t} - \bar{y}_t)^2}{\sigma_t^2}$. Here, $\bar{y}_t$ is the population-averaged drug accumulation measurement at time t; $D_{T,t}$ is the drug accumulation predicted by the model; σ_t is the measurement error calculated based on a coefficient of variation of 4%

(we obtained this from fluorescence measurements of the PDMS background); and the sum runs over all the time-points 0 to 400 s. Minimization was performed using Matlab's in-built nonlinear least-squares solver (lsqcurvefit; with the maximum number of iterations set to 15). To find the global optimum of ϵ, we repeated the minimization task starting from 500 different initial points (generated using a Sobol sequence of quasi-random numbers) covering the entire parameter space.

We analyzed the single-cell data using a Bayesian hierarchical version of the model in which parameters M_0 and v vary between single-cells. In particular, we postulate that these model-parameters are distributed at the population level according to two independent log-normal distributions[11]. Below, M_0' and v' denote the rescaled versions of M_0 and v which accommodate fitting the model to data normalized by the fluorescence of the drug dose (Table S2). The mean $(\mu_{M_0'}, \mu_{v'})$ and standard deviation parameters $(\sigma_{M_0'}, \sigma_{v'})$ of each log-normal distribution dictate the average value of the corresponding model-parameter and its spread across a bacterial population. Posterior estimates of these population parameters $(\mu_{M_0'}, \mu_{v'}, \sigma_{M_0'}, \sigma_{v'})$ were inferred from single-cell data (experimental repeats were treated separately) using Gibbs sampling and informative priors based on the MLE estimates obtained in the step above (see Figures S6, S7 and Table S3). In the first iteration ($j = 1$) of the algorithm, $\mu_{M_0'}^{(1)}, \mu_{v'}^{(1)}, \sigma_{M_0'}^{(1)}$, and $\sigma_{v'}^{(1)}$ were drawn from their corresponding prior distributions and for each cell $i = 1, \ldots, K$ model-parameters $M_{0,i}'^{(1)}, v_i'^{(1)}$ were obtained by minimizing the discrepancy between the model-predicted accumulation profile and the single-cell measurements $\boldsymbol{y}_i = \{y_{i,t}: t = 1, \ldots, Z\}$. Subsequent iterations ($j > 1$) involve sampling in-turn from the full conditionals:

a) $M_{0,i}'^{(j)}, v_i'^{(j)} \sim P\left(\cdot\,|\boldsymbol{y}_i, \mu_{M_0'}^{(j-1)}, \mu_{v'}^{(j-1)}, \sigma_{M_0'}^{(j-1)}, \sigma_{v'}^{(j-1)}\right)$;

b) $\mu_{M_0'}^{(j)}, \mu_{v'}^{(j)} \sim P\left(\cdot\,|\left\{M_{0,i}'^{(j)}, v_i'^{(j)}: i = 1, \ldots, K\right\}, \sigma_{M_0'}^{(j-1)}, \sigma_{v'}^{(j-1)}\right)$;

c) $\sigma_{M_0'}^{(j)}, \sigma_{v'}^{(j)} \sim P\left(\cdot\,|\left\{M_{0,i}'^{(j)}, v_i'^{(j)}: i = 1, \ldots, K\right\}, \mu_{M_0'}^{(j)}, \mu_{v'}^{(j)}\right)$.

In our analysis, we used conjugate priors for $\mu_{M_0'}, \mu_{v'}, \sigma_{M_0'}, \sigma_{v'}$, i.e., normal priors for $\mu_{M_0'}$ and $\mu_{v'}$, and gamma priors for $\sigma_{M_0'}^{-1}$ and $\sigma_{v'}^{-1}$. This choice greatly simplifies steps (b) and (c) as the target sampling distributions are the updated normal and gamma distributions, respectively. In step (a) for each cell i we sampled from the target distribution:

$$P\left(M_{0,i}', v_i'|\boldsymbol{y}_i, \mu_{M_0'}^{(j-1)}, \mu_{v'}^{(j-1)}, \sigma_{M_0'}^{(j-1)}, \sigma_{v'}^{(j-1)}\right) \propto P(\boldsymbol{y}_i|M_{0,i}', v_i')P\left(M_{0,i}', v_i'|\mu_{M_0'}^{(j-1)}, \mu_{v'}^{(j-1)}, \sigma_{M_0'}^{(j-1)}, \sigma_{v'}^{(j-1)}\right)$$

using a single Metropolis-Hasting step with a bivariate normal as the proposal distribution (covariance matrix set to $10^{-4}\mathbf{I}$, where **I** is the 2x2 identity matrix). All results presented were obtained by running the Gibbs sampler for 2000 iterations (after having discarded 500 'warm-up' iterations).

Theoretical predictions from the model of drug transport across the Gram-negative cell envelope:

We rationalized our experimental single-cell drug accumulation data via our mathematical model, where parameters governing porins (M_0) and efflux pumps (v) are allowed to vary between cells in the population according to a log-normal distribution[11]. The inferred parameter distributions for growing bacteria from the three investigated strains are presented in Figure S5A-B; the different experimental repeats are signified by solid, dotted and dashed lines (WT, red; Δ*ompF*, blue; Δ*tolC*, green). We found similar values across the different replicates for the WT cells, whereas the knockout mutants showed

greater variability both between replicates and within individual experiments, as observed in Figure S5A-B. The parameter estimations also confirmed lower porin concentrations in the Δ*ompF* mutant compared to the WT. Note that due to the flatness of the accumulation profiles of the stationary phase cells, we chose not to infer model parameters from those experiments.

Once model parameters were inferred from all the individual experiments (using the corresponding drug dosage profiles for each experiment), we used these parameters in the model to predict drug accumulation in the various subcellular compartments for cells belonging to the three strains (Figure S5C). In this estimation for Figure S5C, we used an average experimental drug dosage profile (dashed black line, top panel, Figure S5C) as the input. The overlap (or lack thereof) between the [20,80] posterior predictive intervals allows us to predict the probability of WT cells having a higher/lower ofloxacin concentration than each of the mutants, at the subcellular level. The pairwise comparisons (at t = 400 s) for the different strains/compartments are presented in Table S4.

The model predicts that the drug saturates all the binding sites in the outer membrane within approximately 175 s in all three strains. The WT strain has the highest outer membrane drug concentration, with the Δ*ompF* mutant having an approximately 2.25-fold lower concentration, which corresponds to the fewer binding sites available in the mutant (Figure S5A). At the end of the experiment, the probability that the WT strain has a higher drug concentration than the Δ*ompF* mutant in the outer membrane is 0.924; in contrast, between the WT and the Δ*tolC* mutant, the probability that the WT has more drug in the outer membrane is 0.525, suggesting no appreciable difference (Table S4).

The periplasm is also predicted to contain approximately 30-fold lower ofloxacin concentrations than the cytoplasm for all three strains at t = 400 s – this is likely due to the binding of the ofloxacin molecules to their targets within the cytoplasm. The model also predicts a lag time of approximately 100 s between drug accumulation in the outer membrane versus drug accumulation in the cytoplasm. In the cytoplasm, the difference between the WT and the mutant strains is less obvious. The model predicts that, at the end of the experiment, the WT strain is 71.9% more likely to have a higher drug concentration in the cytoplasm than the Δ*ompF* mutant (Table S4). Comparing the WT and the Δ*tolC* mutant, the corresponding likelihood is 54.9% (probability of 0.549).

Supplementary References

1. M. Zampieri, M. Zimmermann, M. Claassen and U. Sauer. *Cell Rep.* , 2017, **19**, 1214–1228.
2. H.-R. Park *et al. Photochem. Photobiol.* , 2002, **75**, 237–248.
3. F. Feijó Delgado *et al. PLoS One* , 2013, **8**, e67590.
4. S. Navaratnam and J. Claridge. *Photochem. Photobiol.* , 2000, **72**, 283–290.
5. A. Zarkan *et al. Sci. Rep.* , 2019, **9**, 3868.
6. A. Smith *et al. Front. Microbiol.* , 2018, **9**, 1739.
7. J. Cama *et al. Sci. Rep.* , 2016, **6**, 32824.
8. R. A. Bamford *et al. BMC Biol.* , 2017, **15**, 121.
9. T. A. Richards, R. Massana, S. Pagliara and N. Hall. *Philos. Trans. R. Soc. B* , 2019, **374**, 20190076.
10. J. R. Swedlow, K. Hu, P. D. Andrews, D. S. Roos and J. M. Murray. *Proc. Natl. Acad. Sci. U. S. A.* , 2002, **99**, 2014–2019.
11. C. Furusawa, T. Suzuki, A. Kashiwagi, T. Yomo and K. Kaneko. *Biophysics (Oxf).* , 2005, **1**, 25–31.

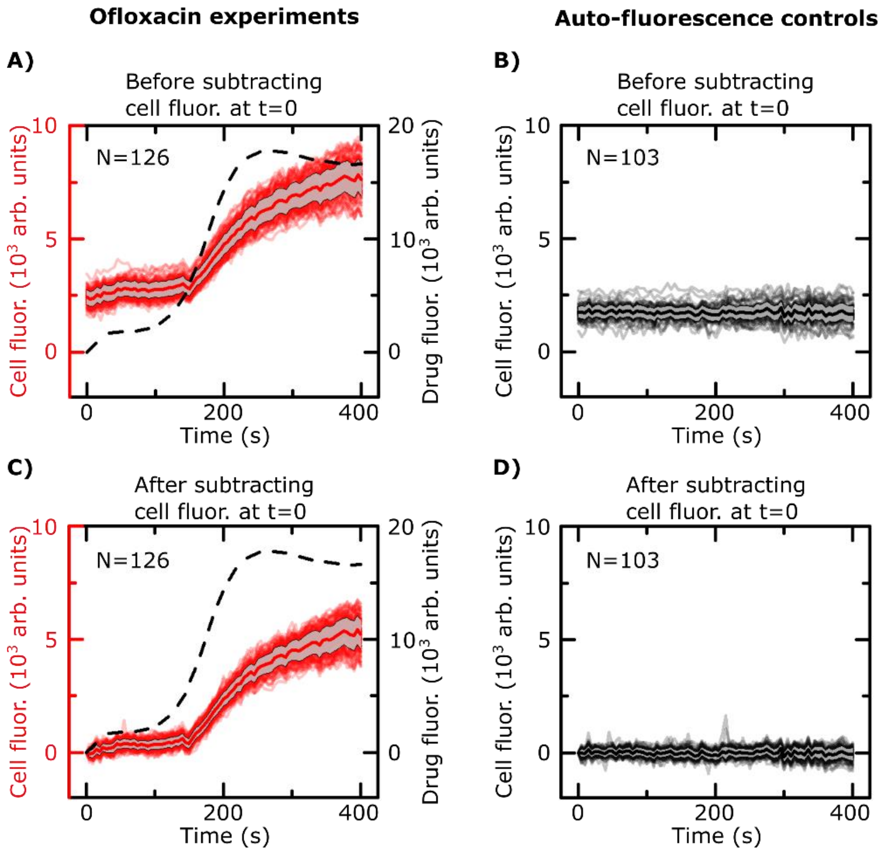

Fig. S1.

Comparison of drug fluorescence (A, C) versus auto-fluorescence control (B, D) cellular profiles of wild type (WT) *E. coli* (growing). In the top panels (A and B), the cellular fluorescence profiles report the fluorescence intensities of individual bacteria after the subtraction of the PDMS background fluorescence as explained in the Experimental section. The bottom two panels (C) and (D) report the fluorescence intensities of the cells in the same experiments as reported in (A) and (B), respectively, after *also* subtracting the initial cell fluorescence values at $t = 0$. In both experiments, the cells were grown on chip for 3 h in fresh LB medium at a flow rate of 100 μl/h, followed by a wash (10 min at 300 μl/h) with 1 g/L glucose dissolved in minimal media prior to dosage with ofloxacin (A, C) or PBS (B, D). The drug fluorescence experiment (A, C) shows the delivery (dashed black line) of ofloxacin in the main channel and corresponding drug accumulation profile of 126 WT *E. coli* cells (red); the mean (thick red line) and standard deviation (grey shading) for the cell profiles are also shown. In the absence of the drug, the cellular auto-fluorescence profiles are flat (B, D; 103 individual cells shown in grey with the mean in black, along with the standard deviation as the grey shaded region). We conclude that cellular auto-fluorescence has a negligible contribution to the drug fluorescence profile of a cell under the conditions of our experiment.

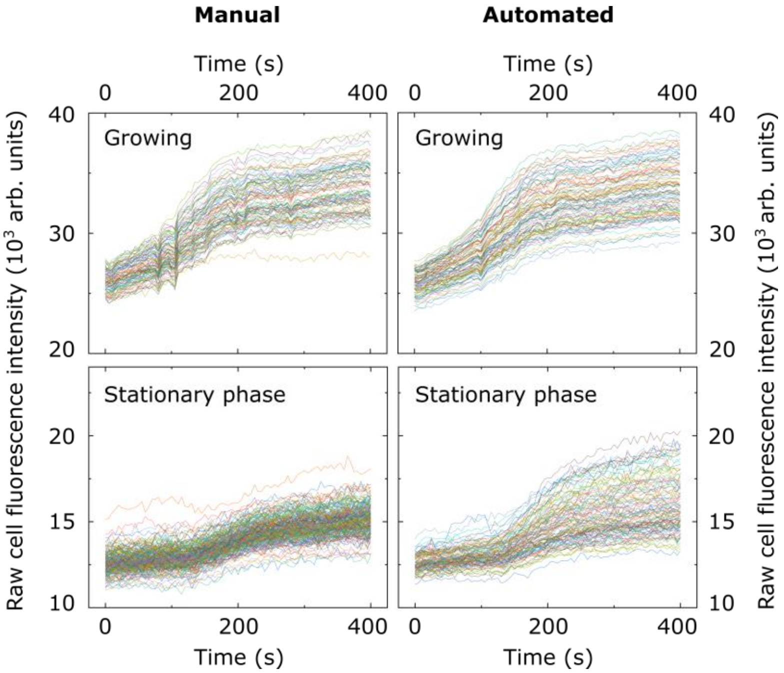

Fig. S2.

Comparison of manual vs automated image analysis of the raw cell fluorescence intensities for *E. coli* (WT) cells exposed to ofloxacin. Note that these plots represent the *raw* fluorescence intensities of individual cells, *before* the (critical) background subtraction. The raw intensities at t=0 s are higher in the "growing" cell experiments, due to the higher background likely caused by the residual fluorescence of LB absorbed by the PDMS; this is accounted for during the background subtraction described in the Experimental section. The plots above compare the tracking of cellular fluorescence in a "growing" experiment (top panels) and a "stationary phase" experiment (bottom panels). From the comparisons, it is clear that the manual and automatic analysis agree very well for growing cells, which are larger than stationary phase cells and hence easier to segment. For a quantitative comparison, the (raw) cell fluorescence intensities (mean ± s.d., arb. units) in the growing cell experiment at 400 s are 33550 ± 2157 (automated) compared to 33691 ± 1989 (manual), showing excellent agreement. As noted in the main text, the segmentation and tracking of the smaller stationary phase cells is more difficult, which is why the automated segmentation reports noisier results than the manual analysis for the stationary phase experiment. However, the mean results are similar within standard deviation, with values of 16080 ± 1609 (automated) and 15203 ± 815 (manual). As mentioned in the text, we do not apply the model to the stationary phase data, and only use the mean values for analysis (after the subtractions and normalization described in the Experimental section).

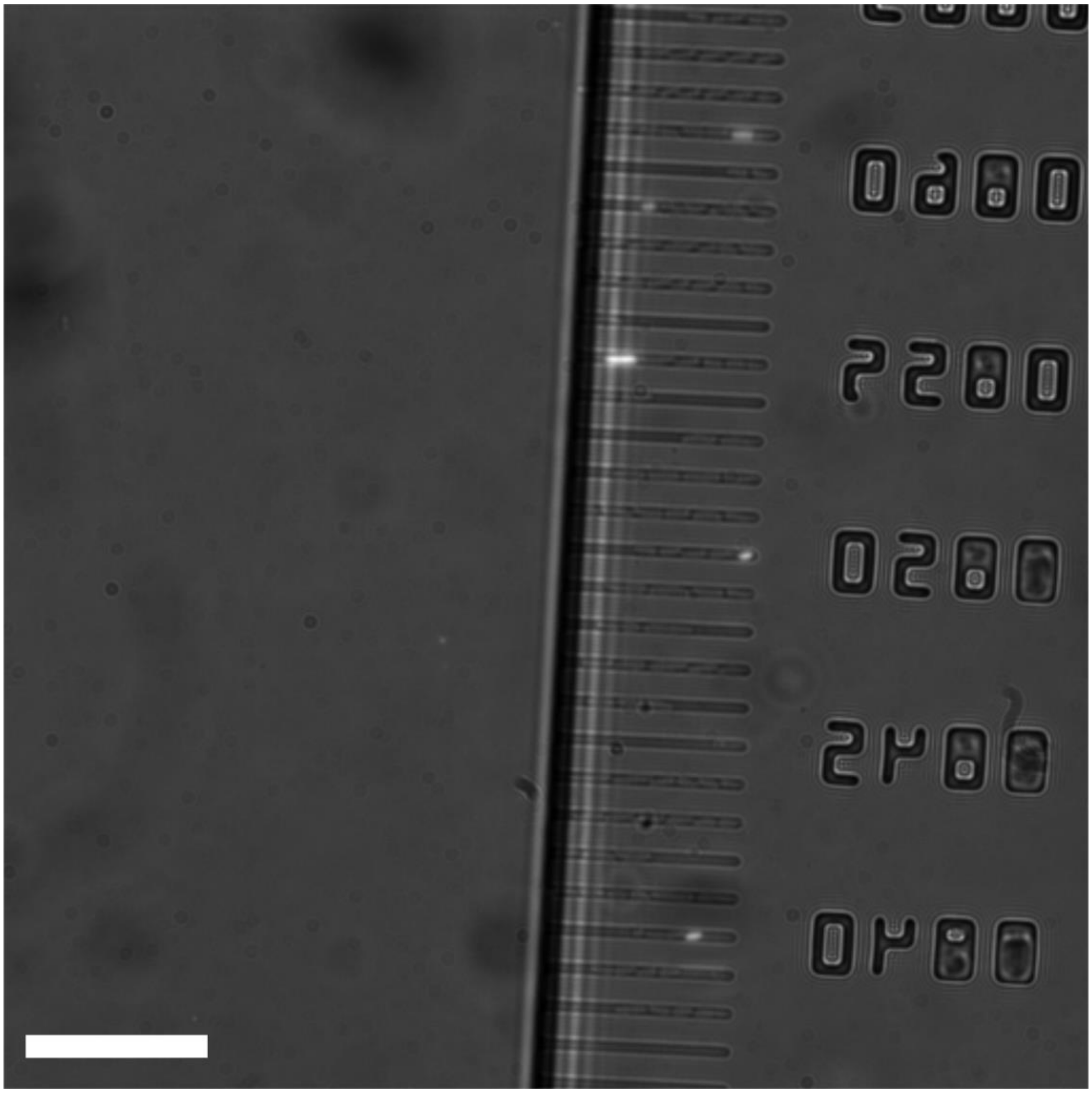

Fig. S3.

Representative combined bright-field and fluorescence image showing the effects of Propidium Iodide (PI) staining on the bacteria post UV and ofloxacin exposure. The PI test shows that cellular membrane integrity remains intact during the drug accumulation experiments. WT *E. coli* cells were grown for 3 h on fresh LB in the chip and treated with UV (λ_{ex} = 365 nm) and ofloxacin as per the standard drug accumulation experimental protocol (Experimental section). After the ofloxacin treatment, the cells were treated with PI for 10 min (flow rate 100 μl/h). Less than 5% of the cells stain with PI; similar levels of PI staining were obtained in cells that did not receive the focused UV treatment. Our results indicate that the UV exposure (λ_{ex} = 365 nm) does not damage cellular membrane integrity for the majority (>95%) of the cells within the timescale of the drug accumulation experiments. Scale bar = 25 μm.

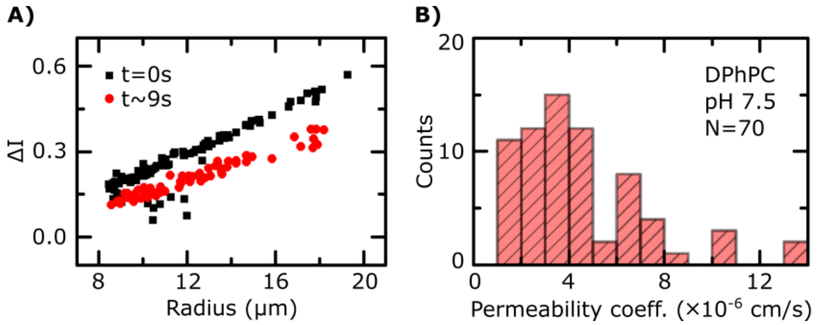

Fig. S4.

Ofloxacin permeability measurement across DPhPC lipid vesicle membranes at pH 7.5. Lipid vesicles were prepared by electroformation and treated with a solution of 2 mM ofloxacin in a T-junction microfluidic chip (for technical details of the measurement conditions and assay, please refer to Cama *et al. Sci. Reps.* 2016). Drug permeation across the vesicle membranes is tracked using the UV auto-fluorescence of ofloxacin. **A)** ΔI refers to a normalized intensity difference between the interior and exterior of the vesicles. The shift in ΔI between vesicles detected at t = 0 (black squares) and those detected at a later time point (t ~ 9 s, red circles) is a direct readout of drug transport into the vesicles, associated with an increase in the fluorescence intensity of the vesicles due to the accumulation of the drug. **B)** Permeability coefficient histograms associated with the measurement of ofloxacin uptake in 70 vesicles. The permeability coefficient of ofloxacin is calculated based on a simple diffusion model (details in Cama *et al. Sci. Reps. 2016*, Cama *et al. Lab Chip* 2014). The permeability coefficient measured was $P = 4.5 \pm 0.3 \times 10^{-6}$ cm/s (N = 70, mean ± s.e.m.).

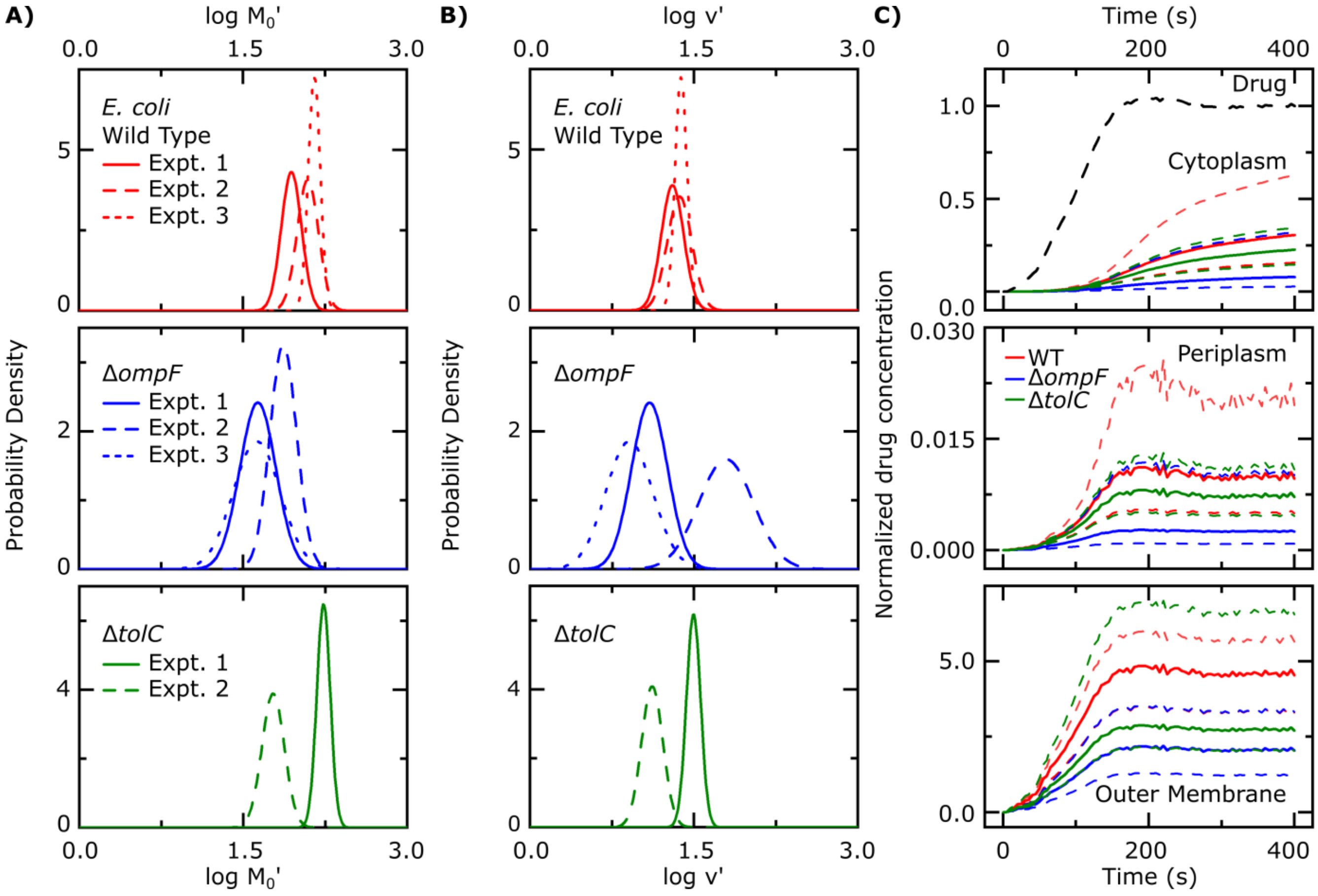

Fig. S5.

Drug accumulation kinetics predicted by fitting the single-cell data to the drug accumulation model. Maximum aposteriori estimates of the population distribution of parameters M_0'**(A)** and v' **(B)** for growing wild type (WT, top), Δ*ompF* (middle) and Δ*tolC* (bottom) *E. coli*. The solid, dashed and dotted lines refer to individual experimental repeats. These distributions were generated using the mode of the joint posterior distribution of the means and standard deviations of the log-normal distributions for M_0' and v'; the marginal posterior distributions of the means and standard deviations for the parameters are provided in Figures S6 and S7 respectively. **C)** Predicted ofloxacin accumulation in the different bacterial compartments. Temporal dependence of the normalized drug concentration in the cytoplasm, periplasm and outer membrane for WT (red), Δ*ompF* (blue) and Δ*tolC* (green) *E. coli* in response to the drug dosage input (dashed black line, top panel). These drug accumulation profiles were obtained by using the kinetic parameter values in (A) and (B) and the theoretical model (equations (i)-(iii)). The concentrations reported are normalized to the drug dosage concentration (12.5 μg/ml ofloxacin). The solid lines correspond to median accumulation in the respective compartments and the dashed lines indicate the [20,80] posterior predictive intervals of the accumulation (note: in the Outer Membrane panel, the lower dashed red line overlaps with the upper dashed blue line, and the lower dashed green line overlaps with the solid blue line). The results shown were generated by running the model using 500 independent samples of parameters M_0' and v' from their joint posterior distributions. All other parameters were fixed to the values given in Table S2. The model predicts the saturation of binding sites in the outer membrane. The median saturation concentration in the outer membrane is approximately 2.25-fold higher in the WT compared with the Δ*ompF* strain. The periplasmic drug concentrations are approximately 30-fold lower than the cytoplasmic concentrations, which is likely due to the drug binding to its targets within the cytoplasm. Using the [20,80] posterior predictive intervals, we have calculated the probabilities of cells from the different strains showing higher/lower accumulation in the different compartments in a pairwise manner. These results are provided in Table S4.

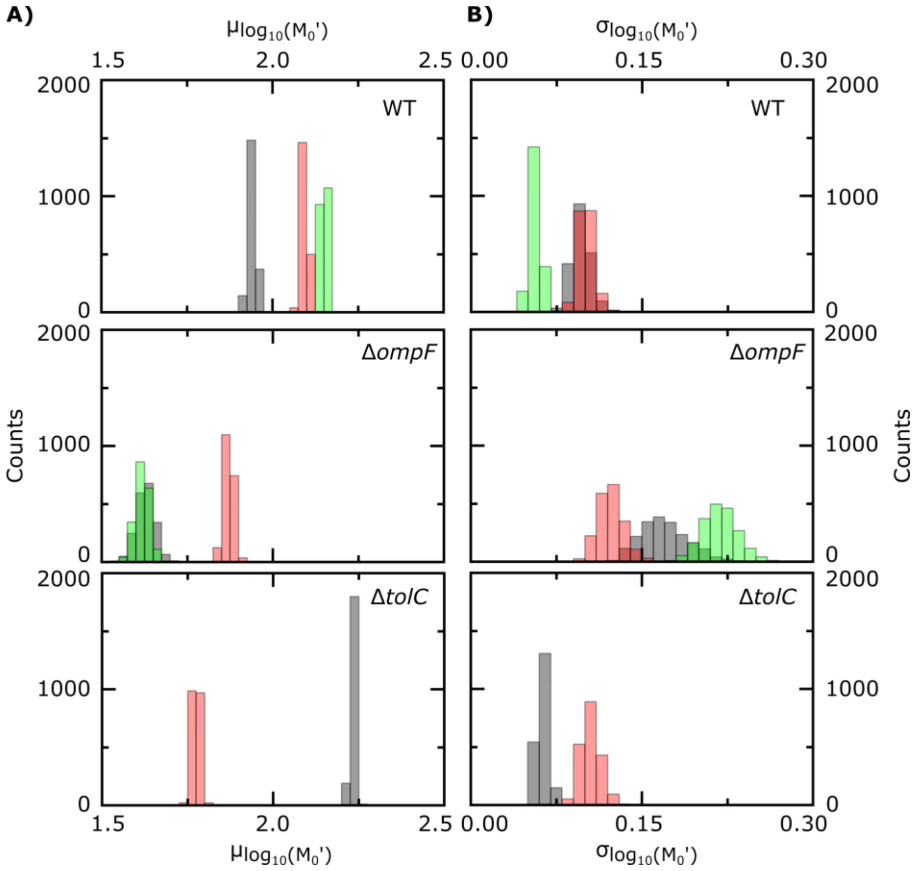

Fig. S6.

Posterior distributions for the means (A) and standard deviations (B) of the log-normal distributions for M_0' in individual experiments. Individual experimental repeats are represented in different colours for each *E. coli* strain.

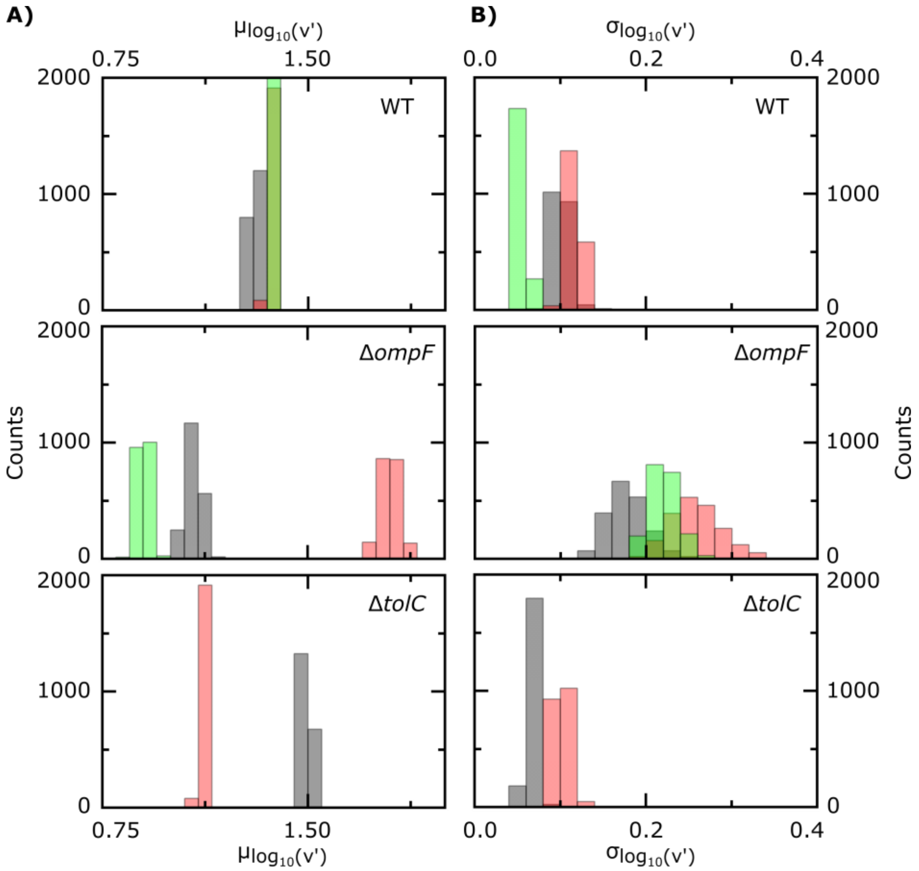

Fig. S7.

Posterior distributions for the means (A) and standard deviations (B) of the log-normal distributions for v' in individual experiments. Individual experimental repeats are represented in different colours for each *E. coli* strain.

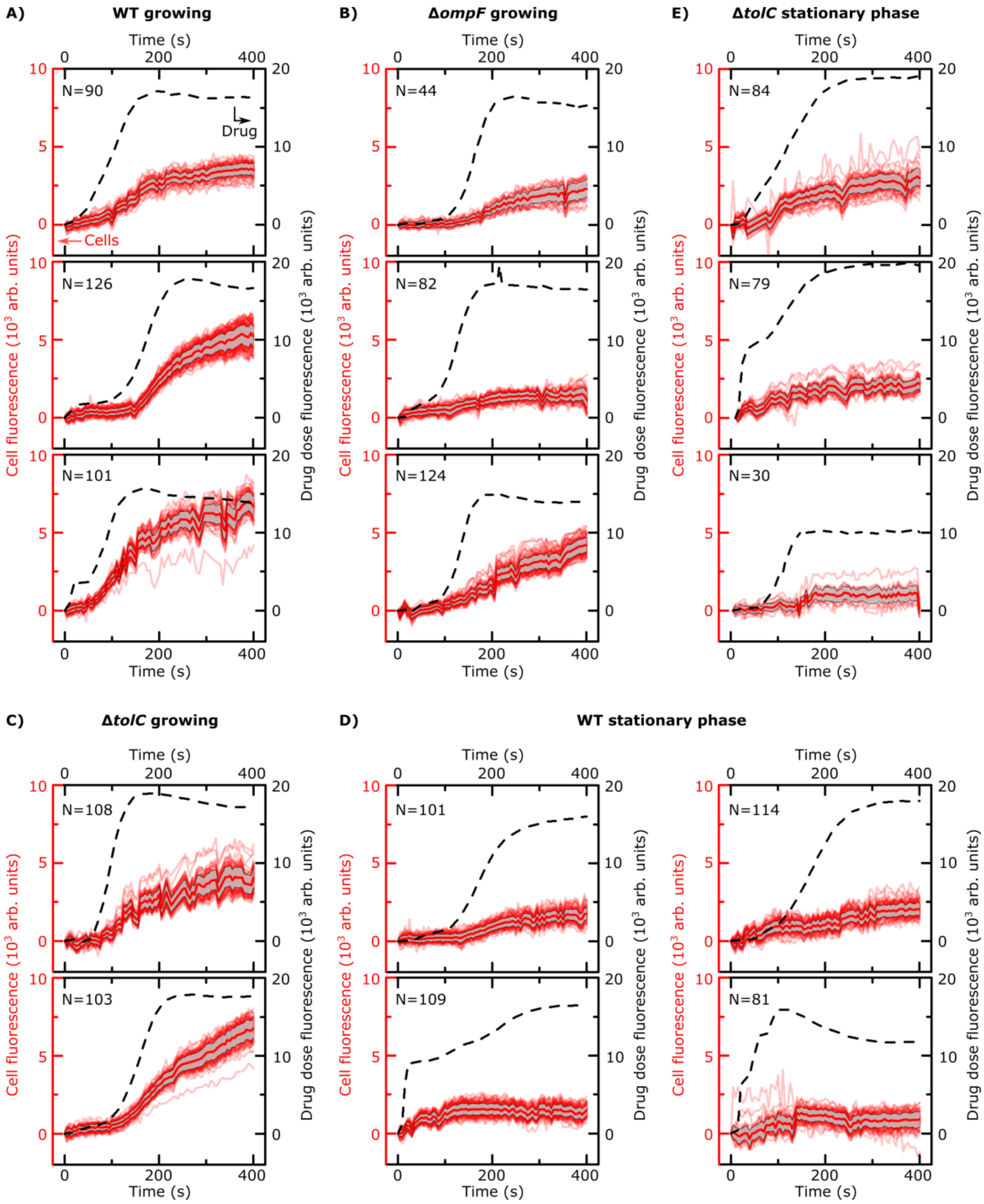

Fig. S8.

Complete dataset showing all experimental repeats for the ofloxacin accumulation experiments. Each plot corresponds to an individual repeat, with the strains/conditions distributed in panels (A)-(E). Black dashed lines correspond to drug dosage profiles (right Y-axes), red lines correspond to cellular fluorescence profiles (left Y-axes) with the mean (thick red line) and standard deviation (grey shading) also shown in the individual plots. N refers to the number of cells in the individual experiments. All values reported after subtracting the corresponding backgrounds and the cell fluorescence at t = 0 (see Experimental section).

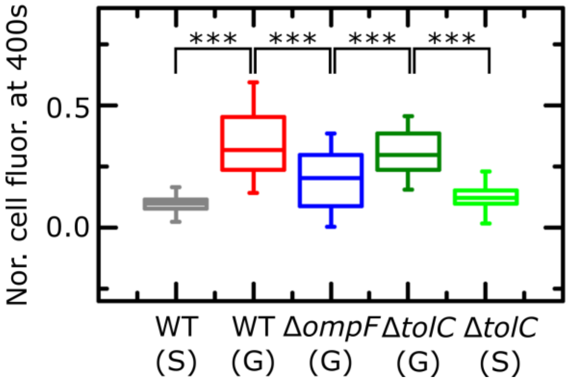

Fig. S9.

Comparison of data pooled from the different experiments shows that stationary phase WT *E. coli* show significantly lower ofloxacin accumulation than growing WT *E. coli* (p<10^{-10}). This was also true in the Δ*tolC* strain, where stationary phase cells showed significantly lower accumulation (p<10^{-10}) than growing cells, suggesting ofloxacin accumulation critically depends on the growth phase of the cells within the timescales of our experiment. Growing Δ*ompF E. coli* showed lower whole cell drug accumulation than growing WT (p<10^{-10}) and Δ*tolC* (p<10^{-10}) cells, in line with expectations. However, growing Δ*ompF E. coli* accumulated *more* ofloxacin than stationary phase WT cells (p<10^{-10}), suggesting that the growth phase of the cells as set by the nutrient environment plays an even more important role than the deletion of *ompF* in drug accumulation. The horizontal lines in the interior of the boxes report the medians of the respective distributions. Statistical significance tested using a 2-sample t-test incorporating Welch's correction; the complete set of p-values is reported in Table S1.

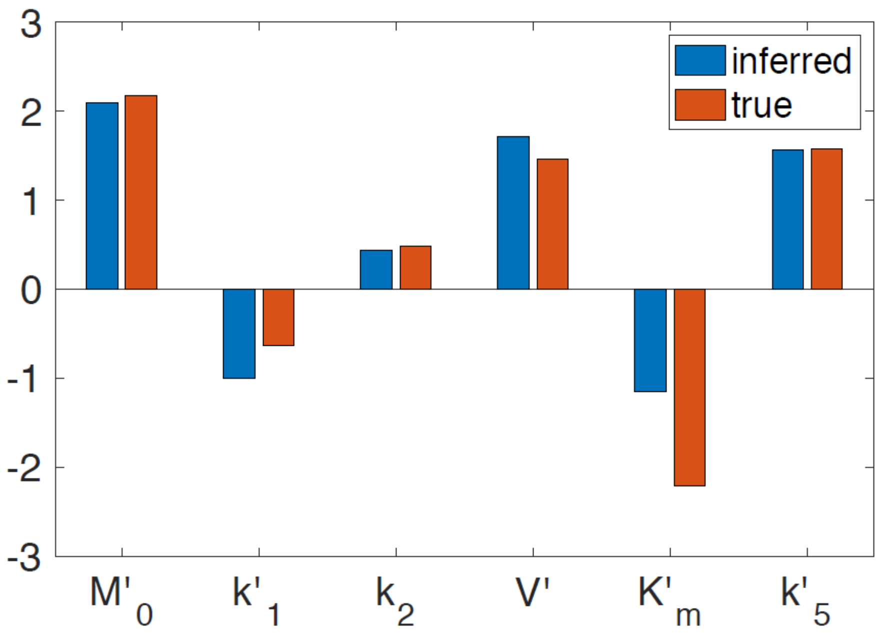

Fig. S10.

Comparison between parameter log₁₀-values inferred from simulated data (inferred, blue) and the actual values used to simulate the data (true, red). We used the model to simulate single-cell drug accumulation profiles similar to those we observed experimentally for WT *E. coli*, and the Δ*ompF* and Δ*tolC* mutant strains. The parameter values we used can be found in Table S2. To introduce cell-to-cell heterogeneity, we assumed that parameters M_0' and v' varied within the different populations according to the distributions found in Figures S5A and S5B. Parameter values were recovered from population-averaged profiles using the methodology described above.

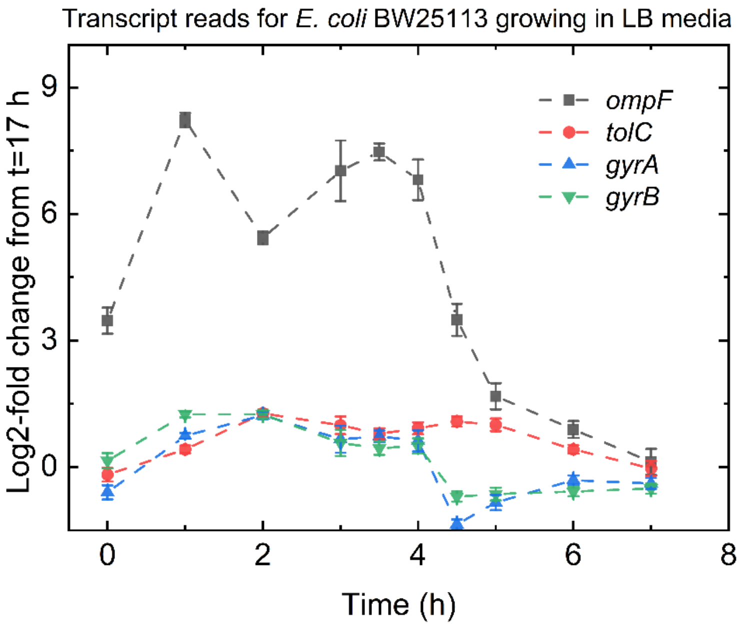

Fig. S11.

Population level transcriptomic data for the *ompF*, *tolC*, *gyrA* and *gyrB* genes of *E. coli* BW25113 (WT strain) grown in LB media (10g/L NaCl). Reproduced using data made available in Smith *et al.*, *Front. Microbiol.* 2018. The time axis refers to the time of growth in LB following the seeding of a stationary phase (17 h of growth in LB) culture into fresh LB. Dashed lines connecting the data points are provided as guides for the eye. Error bars represent the standard error of the mean.

**Comparison of whole cell fluorescence (normalized) of ofloxacin treated cells at *t* = 400 s**	**p-value (2 sample t-test with Welch's correction)**
WT stationary phase vs WT growing	1.4×10^{-123}
WT stationary phase vs Δ*ompF* growing	1.2×10^{-33}
WT stationary phase vs Δ*tolC* growing	1.3×10^{-98}
WT stationary phase vs Δ*tolC* stationary phase	1.9×10^{-5}
WT growing vs Δ*ompF* growing	1.8×10^{-42}
WT growing vs Δ*tolC* growing	2.7×10^{-4}
WT growing vs Δ*tolC* stationary phase	8.5×10^{-112}
Δ*ompF* growing vs Δ*tolC* growing	7.4×10^{-30}
Δ*ompF* growing vs Δ*tolC* stationary phase	2.5×10^{-21}
Δ*tolC* growing vs Δ*tolC* stationary phase	3.2×10^{-92}

Table S1.

Two sample t-test (with Welch's correction) p-values for the whole cell fluorescence (background subtracted, normalized) of the different strains/conditions at *t* = 400 s (Figure S9) in the ofloxacin accumulation experiments. Sample sizes: WT stationary phase (*N* = 405), WT growing (*N* =317), Δ*ompF* growing (*N* = 250), Δ*tolC* growing (*N* = 211) and Δ*tolC* stationary phase (*N* = 193).

Parameter	Units	Value	Notes
k_1	$\text{molecules}^{-1} \cdot \mu m^6 \cdot \text{sec}^{-1}$	1.12×10^{-5}	Inferred from cell data.
k_2	$\mu\text{m}^3 \cdot \text{sec}^{-1}$	3.08	Inferred from cell data.
k_3	$\mu\text{m}^3 \cdot \text{sec}^{-1}$	0.52	Estimated from data in Fig. S4.
k_4	$\mu\text{m}^3 \cdot \text{sec}^{-1}$	0	Assumed negligible.
k_5	$\mu\text{m}^3 \cdot \text{sec}^{-1}$	0.0139	Inferred from cell data.
M_0	$\text{molecules} \cdot \mu m^{-3}$	3.11×10^6	Inferred from cell data.
v	$\text{molecules} \cdot \text{sec}^{-1}$	6.11×10^5	Inferred from cell data.
K_m	$\text{molecules} \cdot \mu\text{m}^{-3}$	1.30×10^2	Inferred from cell data.
V_M	μm^3	0.0565	The bacterial cell is modelled as a cylinder of total width 1 μm. The width of the outer membrane was estimated to be 5 nm, and that of the periplasmic space 30 nm.
V_P	μm^3	0.326	
V_C	μm^3	2.45	
Model fitting			
Parameters	$k_1' = k_1 \cdot A\,; M_0' = \frac{M_0}{A}; v' = \frac{v}{A}; K_m' = \frac{K_m}{A};$ $k_2; k_5' = k_3/k_5$		Parameterization for fitting the model with fluorescence data. $A = 2.08 \times 10^4$ molecules $\cdot \mu m^{-3}$ is the drug dose concentration.
For the estimation of parameters from the population-averaged data, to ease exploration of the parameter space, all parameters above were log (base 10) transformed. The range of parameter $\log_{10} k_5'$ was set to [0,5] to satisfy the constraint $k_5 \leq k_3$. The range of parameter M_0 was constrained in [1.93, 2.23] so that the corresponding count of porins per cell lies between 1×10^5 to 2×10^5. All other parameters were allowed to vary between [-5, 5].			

Table S2.

Model parameter estimates for wild type *E. coli*.

Parameter	Values
$\boldsymbol{\mu}_{\log_{10}(M_0')}$	$\boldsymbol{\mathcal{N}}(\mu = 2.175, \sigma = 0.5)$
$\boldsymbol{\mu}_{\mathbf{log_{10}}(v')}$	$\boldsymbol{\mathcal{N}}(\mu = 1.47, \sigma = 0.5)$
$\boldsymbol{\sigma}_{\log_{10}(M_0')}$	$\Gamma(\alpha = 10^{-4}, \beta = 10^{-4})$
$\boldsymbol{\sigma}_{\mathbf{log_{10}}(v')}$	$\Gamma(\alpha = 10^{-4}, \beta = 10^{-4})$

Table S3.

Prior distributions for population parameters in the Bayesian hierarchical model. Normal distributions with mean $\boldsymbol{\mu}$ and std. dev. $\boldsymbol{\sigma}$ were used as priors for $\boldsymbol{\mu}_{\log_{10}(M_0')}$ and $\boldsymbol{\mu}_{\mathbf{log_{10}}(v')}$. Gamma distributions with shape parameter $\boldsymbol{\alpha}$ and scale parameter $\boldsymbol{\beta}$ were used as priors for $\boldsymbol{\sigma}_{\log_{10}(M_0')}$ and $\boldsymbol{\sigma}_{\mathbf{log_{10}}(v')}$.

Probability	Value
$D_M^{\text{WT}} > D_M^{\Delta ompF}$	0.924
$D_M^{\text{WT}} > D_M^{\Delta tolC}$	0.525
$D_P^{\text{WT}} > D_P^{\Delta ompF}$	0.718
$D_P^{\text{WT}} > D_P^{\Delta tolC}$	0.549
$D_C^{\text{WT}} > D_C^{\Delta ompF}$	0.719
$D_C^{\text{WT}} > D_C^{\Delta tolC}$	0.549
$D_T^{\text{WT}} > D_T^{\Delta ompF}$	0.748
$D_T^{\text{WT}} > D_T^{\Delta tolC}$	0.555

Table S4.

Using the model to compare drug accumulation in different strains after 400 s of drug exposure. The model was used to estimate the probability of higher drug accumulation within WT cells compared to Δ*tolC* and Δ*ompF* mutant cells at the subcellular and whole cell level (M, outer membrane; P, periplasm; C, cytoplasm; T, total). Probability estimates are based on 1000 runs of the model for each strain in which parameters M_0' and v' were drawn from the corresponding population distributions shown in Figure S5.

Movie S1.

Ofloxacin accumulation experiment. The movie shows the delivery of ofloxacin (100×MIC, in PBS) to geometrically confined *E. coli* cells (WT) in the microfluidic device via the main channel; drug arrival is detected via its auto-fluorescence (λ_{ex} = 365 nm) in the main channel. Subsequent drug accumulation in the cells is observed as an increase in the drug fluorescence intensity associated with the cells confined in the side channels. Scale bar = 25 μm.
